# Molecular alterations and tumor suppressive function of the *DUSP22 (Dual Specificity Phosphatase 22)* gene in peripheral T-cell lymphoma subtypes

**DOI:** 10.18632/oncotarget.11930

**Published:** 2016-09-10

**Authors:** Pierre Mélard, Yamina Idrissi, Laetitia Andrique, Sandrine Poglio, Martina Prochazkova-Carlotti, Sabine Berhouet, Cécile Boucher, Elodie Laharanne, Edith Chevret, Anne Pham-Ledard, Andréa Carla De Souza Góes, Véronique Guyonnet-Duperat, Alice Bibeyran, François Moreau-Gaudry, Béatrice Vergier, Marie Beylot-Barry, Jean-Philippe Merlio, David Cappellen

**Affiliations:** ^1^ Institut National de la Santé et de la Recherche Médicale (Inserm) U1053, Universitaire de Bordeaux, F-33076 Bordeaux, France; ^2^ Service de Pathologie, Centre Hospitalier Universitaire de Bordeaux, Hôpital Haut-Lévêque, F-33604 Pessac, France; ^3^ Service de Biologie des Tumeurs-Tumorothèque, Centre Hospitalier Universitaire de Bordeaux, Hôpital Haut-Lévêque, F-33604 Pessac, France; ^4^ Service de Dermatologie, Centre Hospitalier Universitaire de Bordeaux, Hôpital Saint-André, F-33000 Bordeaux, France; ^5^ Instituto de Biologia Roberto Alcantara Gomes, Universidade do Estado do Rio de Janeiro, CEP 20550-013 Rio de Janeiro, Brazil; ^6^ Plateforme de Vectorologie, Unité Mixte de Services (UMS TBM-Core), Centre National de la Recherche Scientifique (CNRS)- Institut National de la Santé et de la Recherche Médicale (Inserm)-Universitaire de Bordeaux, F-33076 Bordeaux, France; ^7^ Biothérapies des Maladies Génétiques et Cancers, Institut National de la Santé et de la Recherche Médicale (Inserm), U1035, Universitaire de Bordeaux, F-33076 Bordeaux, France

**Keywords:** T-cell lymphomas, DUSP22, silencing, mutations, tumor suppressor function

## Abstract

Monoallelic 6p25.3 rearrangements associated with *DUSP22 (Dual Specificity Phosphatase 22*) gene silencing have been reported in CD30+ peripheral T-cell lymphomas (PTCL), mostly with anaplastic morphology and of cutaneous origin. However, the mechanism of second allele silencing and the putative tumor suppressor function of *DUSP22* have not been investigated so far. Here, we show that the presence, in most individuals, of an inactive paralog hampers genetic and epigenetic evaluation of the *DUSP22* gene. Identification of *DUSP22*-specific single-nucleotide polymorphisms haplotypes and fluorescence *in situ* hybridization and epigenetic characterization of the paralog status led us to develop a comprehensive strategy enabling reliable identification of *DUSP22* alterations. We showed that one cutaneous anaplastic large T-cell lymphomas (cALCL) case with monoallelic 6p25.3 rearrangement and *DUSP22* silencing harbored exon 1 somatic mutations associated with second allele inactivation. Another cALCL case carried an intron 1 somatic splice site mutation with predicted deleterious exon skipping effect. Other tested PTCL cases with 6p25.3 rearrangement exhibited neither mutation nor deletion nor methylation accounting for silencing of the non-rearranged *DUSP22* allele, thus inactivated by a so far unknown mechanism. We also characterized the expression status of four *DUSP22* splice variants and found that they are all silenced in cALCL cases with 6p25.3 breakpoints. We finally showed that restoring expression of the physiologically predominant isoform in DUSP22-deficient malignant T cells inhibits cellular expansion by stimulating apoptosis and impairs soft agar clonogenicity and tumorigenicity. This study therefore shows that *DUSP22* behaves as a tumor suppressor gene in PTCL.

## INTRODUCTION

Cutaneous T-cell lymphomas (CTCL) are a wide group of heterogeneous diseases representing approximately 2.6% of all non-Hodgkin lymphomas worldwide. Global genomic approaches recently revealed the existence of recurrent alterations, some having potential diagnosis or prognosis value [1, 2, 3, 4, 5, 6, 7, 8]. Recurrent monoallelic rearrangements of the 6p25.3 locus were identified in peripheral T cell lymphomas (PTCL) [9, 10, 11, 12]. Feldman *et al.,* then our team, showed that these 6p25.3 rearrangements are quite specific of CD30-positive/ALK-negative anaplastic large T-cell lymphomas (ALCL), mainly of cutaneous origin (cALCL) [9, 10, 11, 12], but also occur in transformed/tumor stage mycosis fungoides (T-MF) [11] and in rare lymphomatoid papulosis variants [13]. Systemic ALCL with 6p25.3 rearrangements seem to have better clinical outcomes than cases without rearrangements [14].

Among the 3 genes located in this region, *IRF4*, an oncogene activated in various types of hematological [15, 16, 17, 18, 19, 20] and skin [21, 22] cancers, was initially considered the strongest candidate [10, 11]. Then, in one ALCL case, next generation sequencing identified a t(6;7)(p25.3;q32.3) translocation interrupting the *DUSP22* (*Dual Specificity Phosphatase 22*) gene, encoding a tyrosine/serine/threonine phosphatase [23], within intron 1 [12]. Fluorescence *in situ* hybridization (FISH) showed that 7q32.3 was the partner locus in about 30% of PTCL with 6p25.3 rearrangements. Regardless of the partner (7q32.3, other or unknown), tested PTCL cases with monoallelic 6p25.3 alterations exhibited *DUSP22* down-regulation [12], making it a candidate tumor-suppressor at this locus.

Beside its inhibitory effects on various signaling pathways, such as T-cell receptor and STAT3, and on cell migration [23, 24, 25, 26, 27, 28, 29], little is known about the physiopathological roles of DUSP22. Notably, its implication in oncogenesis was never functionally addressed so far. The status of the second allele of *DUSP22* in PTCL with monoallelic 6p25.3 breakpoints-associated silencing was also not yet studied. Finally, sequence databases indicated the existence of alternative *DUSP22* transcripts predicted to encode carboxy-terminally truncated proteins, but their expression levels and functions were never studied. Our goal was thus to investigate the expression status of *DUSP22* isoforms in normal lymphocytes and PTCL, the mechanism of second allele silencing in PTCL with 6p25.3 rearrangements, and whether *DUSP22* exerted tumor-suppressor properties.

## RESULTS

### The *DUSP22* gene encodes various transcripts which are silenced in cutaneous T-cell lymphomas with monoallelic 6p25.3 rearrangements

**Figure 1:**
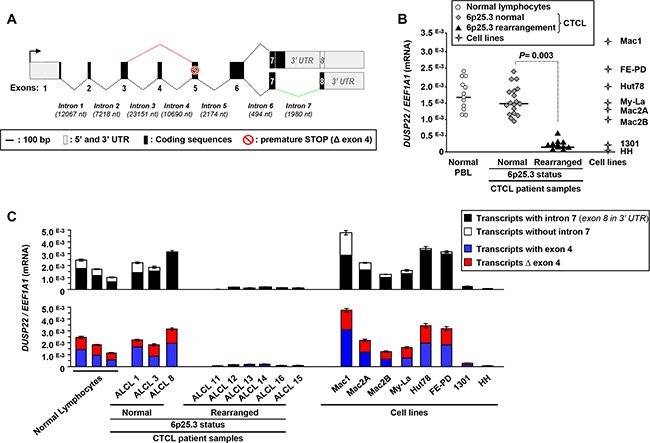
Silencing of *DUSP22* alternative transcripts in cutaneous T-cell lymphomas with monoallelic 6p25.3 breakpoints **A.** Schematic representation of *DUSP22* alternative transcripts. Numbered boxes indicate the exons, with 5′ and 3′ untranslated (UTR) regions in grey and coding region in black. Nt: nucleotides. The black arrow indicates the position of the transcription initiation site. Dotted lines indicate the regular and alternative splicing events (in red, without exon 4; in green, without intron 7). The red symbol highlights the presence of a premature STOP codon which is in frame in Δ exon 4 transcripts. **B.** Quantitative RT-PCR analysis of *DUSP22* transcript levels (forward primer within exon 1 and reverse primer overlapping the junction between exons 6 and 7), normalized by *EEF1A1* expression, in normal peripheral blood leukocytes (PBL), cutaneous T-cell lymphomas cases (CTCL) with or without 6p25.3 rearrangements, and lymphoid T-cell lines. Mean from independent measurements are shown. **C.** *DUSP22* alternatively spliced transcipts levels were analyzed by quantitative RT-PCR, normalized by *EEF1A1* expression, in normal PBL, CTCL cases and lymphoid T-cell lines. Mean ± SEM from independent measurements are shown. For transcripts with and without intron 7 (Top panel), the common forward primer was within exon 1 and isoform-specific reverse primers were either at the beginning of intron 7, or at the beginning of exon 8. For transcripts with and without (Δ) exon 4 (Bottom panel), isoform-specific forward primers were overlapping either exons 3 and 4 or exons 3 and 5, respectively, and the common reverse primer was overlapping the junction between exons 6 and 7.

Four alternatively spliced *DUSP22* gene transcripts were already deposited in databases (Figure 1A, Table 1, Supplementary Figures S1, S2). The transcript encoding the known 184 amino-acids protein [23, 24, 25, 26, 27, 28, 29] comprises 8 exons. An alternative transcript with unspliced intron 7 is predicted to generate a 205 amino-acids protein diverging from the former in the carboxy-terminal region. Exon 4 can be spliced from these two transcripts (Δ exon 4), leading to frameshift-associated premature STOP codon, and a predicted 54 amino-acids carboxy-terminally truncated protein. Expression of these transcripts and their putative proteins had not yet been evaluated in any physiopathological condition.

**Table 1:** SNP haplotypes, methylation and expression/splicing status of *DUSP22* and its paralog

	*DUSP22* (6p25.3)	16p11.2 paralog
***SNPs alleles***		
*rs11242812*	**G**	**A**
*rs1129085*	**G** and/or **A**	**G**
*rs1046656*	**C**	**T**
***Methylation status*** *(5′ CpG island)*	**No methylation**^$^(^$^ *in normal tissues & tested PTCL cases*)	**Methylated**^‡^ (^‡^ *if not deleted in the 5′ region)*
***Expression***	**Expressed in normal tissues**& PTCL without 6p25.3 rearrangement **Silenced in PTCL with 6p25.3 break**	**Completely silent or hypomorphic**
***Isoforms***	**Alternative mRNA splicing**	**Not detected** *(expression absent or very weak)*
	**Regular splicing, 8 coding exons** Protein: 184 a.a. Expressed at the RNA level in PBL Silenced in PTCL with 6p25.3 break	**Not detected**
	**Intron 7 not spliced**, leading to alternative reading frame, exon 8 then being in the 3′ UTR Predicted protein: 205 a.a., p.A170fs37* Predominant in normal PBL Silenced in PTCL with 6p25.3 break	**Not detected**
	**Exon 4 spliced, leading to frameshift** Predicted protein: 54 a.a., p.G47fs9* Expressed at the RNA level in PBL Silenced in PTCL with 6p25.3 break *Not detected at the protein level*	**Not detected**

**Legends:** The SNP haplotypes, methylation and expression status of DUSP22 and its 16p11.2 are summarized here. For isoforms, the splicing events and the predicted consequence at the protein levels (number of amino acids -a. a.- and when there is a alternative reading frame, position of the first amino acid affected by frameshift –fs-, and number of alternative amino acids before the STOP codon -*-). UTR, untranslated region; PTCL, peripheral T-cell lymphomas; PBL, normal peripheral blood lymphocytes.

Here we showed that normal peripheral blood lymphocytes (PBL) predominantly expressed transcripts with unspliced intron 7, over those with intron 7-splicing (Supplementary Figure S1B, Figure 1C, Table 1). Δ exon 4 transcripts, predicted as candidates to nonsense-mediated decay, were readily detected in normal (PBL) and actually expressed equivalently than transcripts with exon 4 (Supplementary Figure S1B, Figure 1C, Table 1).

Other deposited transcripts originating from alternative initiation sites were not detected in the tested normal PBL, cutaneous T-cell lymphomas (CTCL) and cell lines, and not studied further.

Consistent with observations of Feldman *et al.* [12], quantitative RT-PCR showed that, as compared with normal PBL, all cALCL and T-MF cases (Supplementary Table S1) with monoallelic 6p25.3 breakpoints [11] exhibited *DUSP22* silencing (Figure 1B). Alternatively spliced *DUSP22* transcripts were all silenced in CTCL with 6p25.3 alterations (Figure 1C, Table 1, Supplementary Tables S1 and S2), while there was no significant effect on the expression of the neighboring *IRF4* and *EXOC2/SEC5* genes (Supplementary Figure S3A).

### Lack of *DUSP22* deletion or methylation in CTCL with monoallelic 6p25.3 rearrangements

We next looked for inactivating mutations, deletions or methylation-mediated silencing [30] of *DUSP22* in PTCL with 6p25.3 rearrangements. Such rearrangements were previously found monoallelic and there was no deletion of the second allele [10, 11, 12]. As FISH probes used then were mainly flanking the gene [10, 11, 12], we looked for interstitial deletions using probes encompassing *DUSP22* (Supplementary Figure S4A).

**Figure 2:**
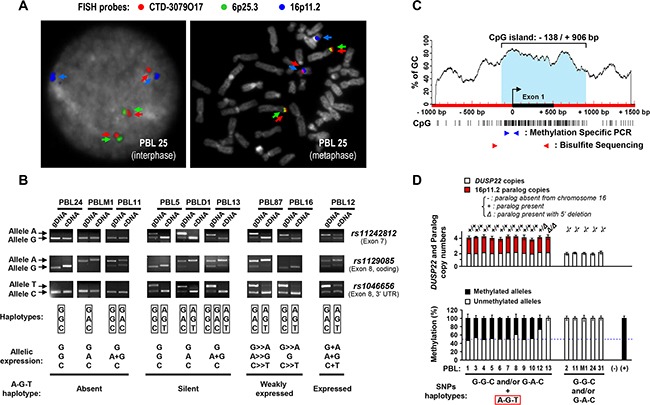
Mapping and haplotype identification of a transcriptionally inactive paralog of the *DUSP22* gene on 16p11.2 **A.** Metaphase and interphase *FISH* analysis of normal peripheral blood lymphocytes (PBL) with a 6p25.3-specific probe (RP11-164H16, Spectrum Green labeled), a probe encompassing the *DUSP22* gene (CTD-3079O17, Spectrum Red labeled), and a 16p11.2-specific probe (RP11-488I20, Spectrum Gold labeled, here displayed in blue (pseudocolor) as colocalization of signals from the former probes may appear in yellow). DAPI (4′,6-diamidino-2-phenylindole dihydrochloride), here visualized in grey (pseudocolor), was used to stain nuclear DNA and chromosomes. Cases exhibiting, in addition to 6p25.3, 1 or 2 additional CTD-3079O17 probe signals, illustrate the existence of a *DUSP22*-related sequence subjected to copy number variations on the 16p11.2 locus (See also Supplementary S5A, B). **B.** Genotype (gDNA, genomic DNA) and allelic expression status (cDNA, complementary DNA) of SNPs *rs11242812*, *rs1129085* and *rs1046656* in representative cases of normal PBL, analyzed by PCR and restriction enzyme digestion (*Msp*I, *Msp*I and *Bss*SI, respectively). Arrows indicate each SNP allele. SNPs genotypes and allelic expression status are summarized for each case under the electrophoresis profiles. **C.** Schematic representation of the CpG island (vertical bars indicate CpG sites) encompassing the 5′ region of the *DUSP22* gene. The black box and curved arrow indicate exon 1 and transcription initiation site, and blue and red arrowheads the primers used for methylation specific PCR (MSP) and bisulfite genomic DNA sequencing (BGS) analyses. **D.** **Top panel**: *DUSP22* and paralog copy numbers, determined by quantitative analysis of SNP *rs1046656* (allele C= *DUSP22*; allele T= paralog), in normal PBL. *KLK3* (19q13.41) was used as a control gene for normalization. Data were matched with results from3 color FISH (discriminating *DUSP22* -6p25.3- from its paralog -16p11.2-, Figure 2A, Supplementary Table S6). The occurrence of deletion in the 5′ region of the paralog was evidenced by the observation of altered qPCR signal ratios between exon 1 and exon 6. **Bottom panel**: Quantitative MSP analysis of *DUSP22/*paralog 5′ CpG island in normal PBL according to the absence or presence of paralog alleles, determined as described above. No DNA (-) and *in vitro* methylated DNA (+) were used as negative and positive controls. Percentages of unmethylated and methylated alleles (Mean ± SEM from independent measurements) are shown.

Consistent with bioinformatics predicting the existence of a paralog of *DUSP22* on 16p11.2 [31, 32], these probes gave, apart from the 6p25.3 locus, additional hybridization signals on 16p11.2 (Figure 2A, Supplementary Figures S4C and S5A).

The presumed high sequence similarity between *DUSP22* and its paralog [31, 32] might alter the interpretation of genetic and epigenetic analyses of *DUSP22*. We thus developed a 3 color FISH approach combined with molecular analyses enabling to distinguish *DUSP22* and its paralog.

Using FISH, we showed that the 16p11.2 paralog was present in most individuals (on one or both chromosomes 16) and absent in ≈10% of cases (Figure 2A, Supplementary Figure S5A, Supplementary Table S6 and Supplementary text file).

Genotyping SNPs *rs11242812*, *rs1129085* and *rs1046656*, we identified specific haplotypes discriminating *DUSP22* (G-G-C and G-A-C alleles) from the 16p11.2 paralog (A-G-T allele) (Figure 2B, Table 1, Supplementary Figure S2, Supplementary Table S6 and Supplementary text file).

Analyzing these SNPs, we next found that, in normal cells, the 16p11.2 paralog was silent, while *DUSP22* was always bi-allelically expressed (Figure 2B, Table 1, Supplementary Figures S6A, S6B, Supplementary Table S6 and Supplementary text file). The paralog was also not expressed in tested CTCL samples.

Finally, consistent with its lack of transcriptional activity, we showed that, while the 5′ CpG island of the *DUSP22* gene was never methylated, the 16p11.2 paralog was mainly methylated (≈80% of alleles) or deleted in that region (Figures 2C, 2D, Table 1, Supplementary Figures S5 and S6, Supplementary Table S6 and Supplementary text file).

**Figure 3:**
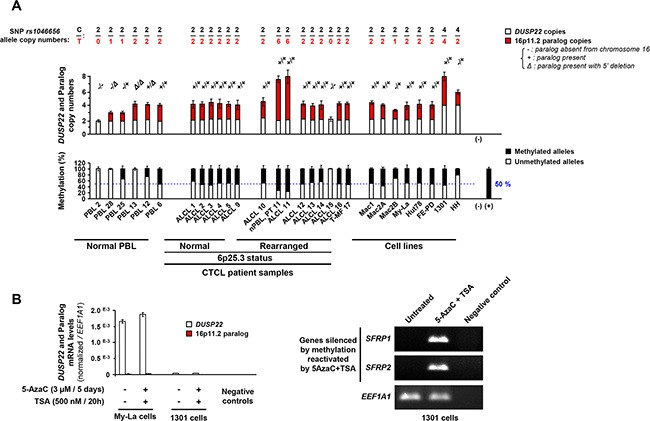
Copy number and methylation status of *DUSP22* and its 16p11.2 paralog in anaplastic large T-cell lymphomas **A.** **Top panel**: *DUSP22* and paralog copy numbers, determined by quantitative analysis of SNP *rs1046656* (allele C= *DUSP22*; allele T= paralog), in normal lymphocytes (PBL) and cutaneous T-cell lymphomas cases (CTCL) and lymphoid T-cell lines. *KLK3* (19q13.41) was used as a control gene for normalization. The occurrence of deletion in the 5′ region of the paralog was evidenced by the observation of altered qPCR signal ratios between exon 1 and exon 6. PBL cases with different 16p11.2 paralog status (2: no copy; 28: one copy with 5′ deletion; 25: one copy; 13: two copies with 5′ deletion; 12: two copies, one having 5′ deletion; 6: two copies) were used as controls. For ALCL case 10, found to carry 6 copies of the 16p11.2 paralog, analysis of the patient's normal PBL (Normal, PT 10) showed that this was a constitutional feature. **A.** **Bottom panel**: Quantitative methylation specific PCR (MSP) analysis of 5′ CpG island present in *DUSP22* and most 16p11.2 paralog alleles, in normal PBL and CTCL samples and cell lines. No DNA (-) and *in vitro* methylated DNA (+) were used as negative and positive controls. Percentages of unmethylated and methylated alleles (Mean ± SEM from independent measurements) are shown. For ALCL case 10, found to constitutionally carry 6 copies of the 16p11.2 paralog (**Top panel**), comparative analysis with the patient's normal PBL (Normal, PT 10) showed that the high level of methylation observed in that tumor is attributed to the paralog copies. **B.** **Left panel**: *DUSP22* and paralog transcript levels were analyzed by allele specific (SNP *rs1046656*: C = *DUSP22*; T = paralog) quantitative RT-PCR, normalized by *EEF1A1* expression, in lymphoid T-cell lines treated or not with 5-Aza-2′-deoxycytidine (DNA methyltransferase inhibitor) trichostatin A (histone deacetylase inhibitor). Mean ± SEM from independent measurements are shown. Such treatment did not significantly alter *DUSP22* expression and notably did not upregulated expression in the 1301 cell line with low levels. **Right panel**: as a control of drug efficiency, *SFRP1* and *SFRP2* genes, known to be silenced by promoter methylation in various cancers, were reactivated by combined treatment with 5-Aza-2′-deoxycytidine and trichostatin A.

Knowledge of the paralog status and identification of *DUSP22*-specific haplotypes allowed comprehensive analysis of *DUSP22* copy number and methylation status in normal PBL and CTCL. Except cases 15 (without paralog) and 11 (six methylated paralog copies), all tested cALCL carried two copies of the methylated and inactive paralog (Figure 3A). In our series of cALCL cases, we found neither *DUSP22* gene deletion (Figure 3A, Top panel) nor *DUSP22* 5′CpG island methylation (Figure 3A, Bottom panel). When detected in tumors, methylation was always attributable to the presence of the paralog (Figure 2D). Indeed, methylation levels were identical in tumors and matching constitutional DNA and proportional to paralog copy numbers (Figure 3A).

### Identification of *DUSP22* somatic mutations in cALCL cases

Further searching for a second hit, we analyzed tumor DNA samples for *DUSP22* mutations in the promixal promoter, exons and splice sites. This screening was performed on 20 cALCL and 19 T-MF, another type of CTCL exhibiting *DUSP22* rearrangements [11] (Supplementary Table S1).

We identified somatic mutations (absent in the constitutional DNA from the same patient) in 2 out of 20 tested cALCL cases (10%), but in none of the T-MF cases.

**Figure 4:**
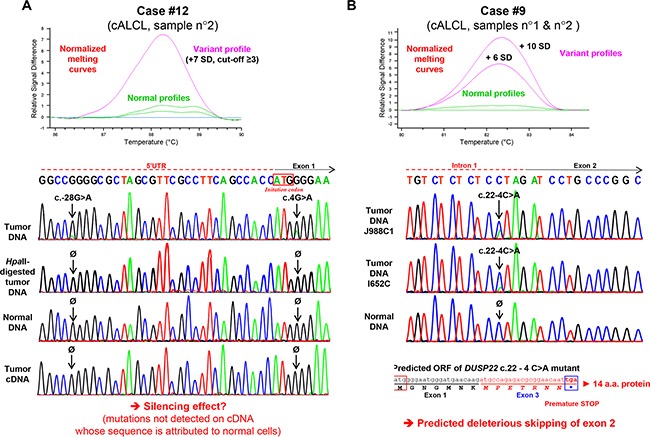
Identification of somatic *DUSP22* mutations in cutaneous anaplastic large T-cell lymphomas **A.** **Top panel**: High resolution melting (HRM) profile of exon 1 genomic DNA PCR products from normal samples and cutaneous anaplastic large T-cell lymphoma (cALCL) case 12, known to harbor monoallelic 6p25.3 breakpoint and *DUSP22* gene silencing. **Bottom panel**: Sanger sequencing analysis of case 12 constitutional and tumor genomic DNA (the latter being digested or not with the *Hpa*II methylation-sensitive restriction endonuclease), as well as tumor complementary DNA (cDNA, obtained from RNA by reverse transcription-PCR). **B.** **Top panel**: High resolution melting (HRM) profile of genomic DNA PCR products encompassing exon 2 and flanking splice sites from normal samples and cutaneous anaplastic large T-cell lymphomas (cALCL) case 9. **Bottom panel**: Sanger sequencing analysis of case 9 constitutional and tumor genomic DNA, with predicted effect of the identified mutation on messenger RNA splicing.

The first case (#12, Supplementary Table S1) harbored a missense c.4G>A nucleotide change immediately after the ATG translation initiation codon, predicted to result in a p.Gly2Arg (p.G2R) amino-acid substitution. It concomitantly exhibited, with minor allele frequency, a c.-28G>A nucleotide variation in the 5′UTR, 28 base-pairs upstream of the initiating codon (Figure 4A). Given the very high estimated homology (99.9%) between *DUSP22* and its paralog [31, 32], present as two methylated copies in this case (Figure 3A, case 12), the PCR primers amplified exon 1 from both *DUSP22* and the paralog. We then digested the tumor DNA with the methylation-sensitive *Hpa*II restriction enzyme, thus fragmenting unmethylated *DUSP22* alleles but not restriction-resistant methylated paralog alleles. Subsequent PCR amplification and sequencing showed only wild-type sequences (Figure 4A), indicating that the mutations affected *DUSP22* and not its paralog. As this case also harbored monoallelic 6p25.3 rearrangement with nearly complete *DUSP22* silencing (Supplementary Table S1 and Figure 1), one or both of these mutations may inactivate the second allele. Sequencing of the weakly expressed tumor *DUSP22* cDNA revealed only wild-type sequences (Figure 4A) attributed to normal cells, indicating that mutant alleles were not expressed, consistent with a silencing effect of these mutations.

Another case (#9, Supplementary Table S1) harbored a c.22-4C>A somatic intronic mutation, 4 base-pairs upstream of exon 2, predicted to lead to exon 2 skipping, with deleterious frameshift effect (Figure 4A). Mutant allele frequency and quantitative SNP analysis were consistent with a heterozygous mutation and the second allele of *DUSP22* was neither rearranged nor deleted (Supplementary Table S1). For this case, frozen material was not available and RNA levels and exon skipping could not be evaluated.

### Characterization of *DUSP22* genetic and expression status in lymphoid T-cell lines

To identify relevant models to study the role of *DUSP22* in oncogenesis, we investigated CTCL cell lines and two other lymphoid T-cell lines (Supplementary Table S1) [33, 34, 35, 36, 37, 38, 39]. Unlike CTCL tumors, none of these 8 cell lines harbored a break-apart FISH pattern with probes flanking *DUSP22* (Supplementary Figure S7A). FISH and high resolution array-comparative genomic hybridization [5] indicated that, in the FE-PD cell line, a 13 Mb region downstream of *DUSP22* was duplicated on 9qter (Supplementary Figure S7A, S7B). FE-PD and most cell lines expressed *DUSP22* as normal PBL, except HH and 1301 which exhibited very weak *DUSP22* transcripts levels (Figure 1B, 1C, Supplementary Figure S1B), though not harboring 6p25.3 rearrangements (Supplementary Figure S7A). The 1301 and HH cell lines, both nearly tetraploid, carried 4 copies of *DUSP22* and respectively 4 and 2 copies of the 16p11.2 paralog, all paralog alleles being methylated in both cell lines (Figure 3, Supplementary Figure S7C). Neither deletion, nor mutations nor 5′ CpG island hypermethylation of *DUSP22* could account for the weak transcript levels observed in the 1301 and HH cell lines, methylation being attributable only to the paralog and proportional to its copy number (Figure 3, Supplementary Figure S7C). Further proving that methylation was not involved in *DUSP22* silencing in these cell lines, there was no reactivation of its expression upon combined 5-aza-2′-deoxycytidine (inhibitor of DNA methyltransferases)/Trichostatin A (inhibitor of histone deacetylases) treatment (Figure 3B, left panel). As a control, such treatment reactivated expression of *SFRP1* and *SFRP2* genes known to be silenced by methylation in various types of cancers (Figure 3B, right panel).

### *DUSP22* exerts tumor suppressive functions

The 205 amino-acids isoform is encoded by the transcript with unspliced intron 7, which is predominant in normal PBL and silenced in PTCL with 6p25.3 rearrangements (Figure 1A, 1C, Table 1, Supplementary Figure S1). This was the only isoform detected at the protein level in normal PBL and cell lines expressing endogenous *DUSP22* transcripts (Supplementary Figure S8A).

**Figure 5:**
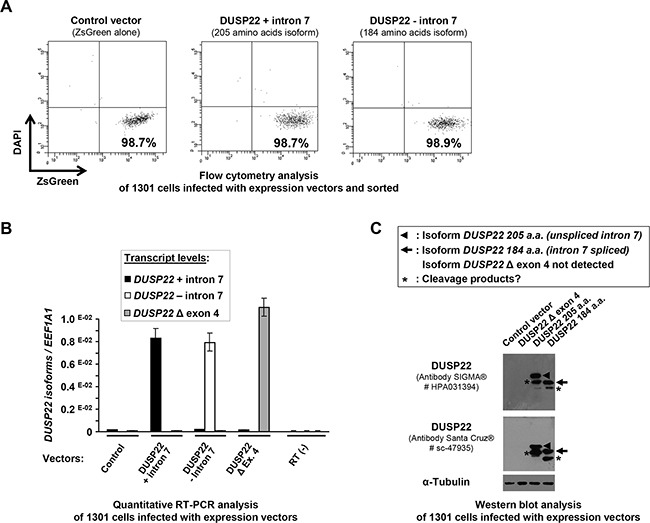
Cell sorting after lentiviral infection and analysis of ectopic expression of *DUSP22* isoforms in 1301 lymphoid T cells **A.** 1301 cells were transduced with lentiviral vectors (Control -encoding the ZsGreen reporter alone- and bicistronic vectors encoding DUSP22 isoforms together with ZsGreen). Alive (DAPI-) and transduced (ZsGreen+) cells were sorted by flow cytometry using ARIA II cell sorter. Dot plots show cell fraction purity after sorting. **B.** *DUSP22* isoforms transcript levels were analyzed by quantitative real-time reverse transcription-PCR (qRT-PCR) on RNA isolated from the same cells transduced with either control or DUSP22 isoforms expression vectors and sorted. *DUSP22* isoform-specific primers were used (Figure 1C and Supplementary Table S2) and transcript levels were normalized for *EEF1A1* gene expression and plotted at the y axis. Mean ± SEM from independent measurements are shown. **C.** Western blot analysis of DUSP22 expression in 1301 cells infected with the control and DUSP22 isoforms vectors and sorted. The anti-DUSP22 antibodies used were from SIGMA® (# HPA031394, as in (A)) and Santa Cruz® (# sc-47935), the latter being directed against an N-terminal peptide common to the 3 isoforms. Alpha-tubulin (α-Tubulin) was used as a control for protein loading.

Western blot analysis of cells transduced with DUSP22 lentiviral expression vectors revealed bands with estimated molecular weight corresponding to the ectopically expressed 205 and 184 amino-acids isoforms (Figure 5C, Table 1, Supplementary Figures S1 and S8D). The Δ exon 4 isoform was never detected, even using an antibody directed against an N-terminal epitope conserved in the DUSP22 isoforms (Bottom panels in Figure 5C and Supplementary Figure S8D), suggesting that this isoform is weakly stable.

Functional studies were thus performed with the 205 amino-acids isoform to investigate the potential tumor suppressor role of *DUSP22* in T-cells. The HH and 1301 cell lines were selected as they exhibited a very low expression of *DUSP22* transcripts and undetectable protein levels (Figure 1B, 1C and Supplementary Figure S8A). Conversely, FE-PD cells were chosen as a control given their high endogenous expression of DUSP22 protein (Supplementary Figure S8A).

FE-PD and 1301 cell lines were very efficiently infected by our lentiviral vectors (95% and 80%, respectively), whereas HH cells could not (as reflected by a percentage of ZsGreen positive cells below 5%) and were thus not further studied. FE-PD and 1301 living infected cells were sorted by flow cytometry prior to functional analyses and checked for DUSP22 expression at the RNA and protein levels (Figure 5, Supplementary Figure S8).

**Figure 6:**
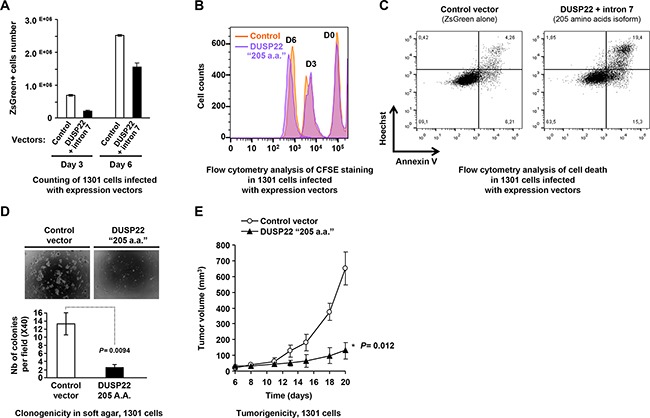
Tumor suppressive effects of DUSP22 in lymphoid T cells **A.** Effect of ectopic expression of the main DUSP22 isoform detected in lymphoid cells on proliferation and viability of 1301 (T cell-acute lymphoblastic leukemia-T-ALL-) cells. Cells infected with either the control (empty) or the DUSP22 205 amino acids (a.a.) isoform (Figure 1A and Supplementary Figure S1) lentiviral vectors and sorted were diluted in equal numbers and grown in the presence of 10% fetal calf serum and counted at different time points. Mean ± SEM from a representative experiment are shown. **B.** Proliferation assay of 1301 cells transduced with either the control (empty) or the DUSP22 205 amino acids (a.a.) isoform lentiviral vectors and sorted. Cells were labeled with carboxyfluorescein succinimidyl ester (CFSE) at day-0 (D0) and its dilution was followed at day-3 (D3) and day-6 (D6) of culture. **C.** Apoptosis and necrosis analyses were performed on infected and sorted 1301 cells by Annexin V/Hoescht 333542 staining 3 days after replating. Single Hoescht 333542-positivity indicates necrotic cells, while double Annexin V/Hoescht positivity hallmarks apoptotic cells. **D.** 1301 T-ALL cells transduced with either the control or DUSP22 (205 a.a. isoform) vectors were assayed for clonogenicity in soft agar. Upper panels: Visualization of colonies using phase contrast microscopy. Bottom panels: Colonies counting plotted as mean ± SEM from independent measurements. **E.** Tumorigenicity of 1301 T-ALL cells, transduced with either the control or DUSP22 (205 a.a isoform) vectors and grafted in immunodeficient mice. Tumor growth is shown for a representative experiment (mean ± SEM from 5 different mice for each condition at different time points).

Ectopic expression of the DUSP22 205 amino-acids isoform had no effect on proliferation of DUSP22-proficient FE-PD cells, and only weakly decreased their viability by slightly enhancing apoptosis (Supplementary Figures S8, S9). In 1301 cells, showing low endogenous DUSP22 levels, lentivirus-mediated expression of this DUSP22 isoform significantly impaired the cell population increase. There was no significant inhibition of cell proliferation but a 5-fold enhancement of apoptosis (Figure 6A–6C).

FE-PD cells neither produced colonies in soft agar, as reported previously [34], nor formed tumors after subcutaneous injection in immunodeficient mice, preventing their use in further functional studies. Remarkably, lentivirus-mediated expression of the physiological DUSP22 205 amino-acids isoform strongly inhibited the clonogenicity of 1301 cells (Figure 6D) and also impaired their tumorigenic properties in a subcutaneous xenograft model (Figure 6E). Altogether, our study shows that inactivation of DUSP22 contributes to T cell transformation and supports a tumor suppressor function of DUSP22.

## DISCUSSION

The silencing of *DUSP22* in PTCL with 6p25.3 rearrangements [12] pointed out this gene as a candidate tumor suppressor whose inactivation may contribute to the pathogenesis of PTCL subtypes, notably ALCL (essentially cutaneous) and T-MF [9, 10, 11, 12, 13, 14]. However, in our cALCL and T-MF cases, 6p25.3 chromosomal alterations were always found to be monoallelic. We thus looked for interstitial deletions or methylation that could account for silencing of the second allele of *DUSP22*. During the course of this analysis, we cytogenetically and molecularly characterized a paralog, mapped on 16p11.2, recently predicted by bio-informatics analyses [31, 32].

This paralog presumably exhibited 99.9% sequence similarity with *DUSP22* [31, 32]. Nonetheless, we could design a strategy combining 3 colors FISH, SNP genotyping and methylation analyses enabling to distinguish *DUSP22* (6p25.3) from its paralog (16p11.2) and allowing comprehensive genetic and epigenetic analyses of *DUSP22* in normal cells, tumors and cell lines.

We showed that this paralog is present in 90% of individuals, on one (30%) or both (60%) 16p11.2 alleles. We also identified *DUSP22*-specific SNP haplotypes and showed that this paralog is either methylated or deleted in the 5′ region and transcriptionally inactive.

CTCL cases with mono-allelic 6p25.3 rearrangements exhibit neither deletion nor methylation of the second allele of *DUSP22*. Methylation of the 5′ CpG island was indeed always linked to the presence of the inactive paralog, except when deleted. The paralog was silent in tested CTCL tumors and cell lines indicating that it is not involved in CTCL pathogenesis.

The above-described strategy can avoid potential misinterpretation of *DUSP22* status. This was first illustrated in 2 CTCL cases (number 15 and 18, Supplementary Table S1) initially thought to exhibit focal heterozygous deletions of *DUSP22* based on previous high resolution comparative genomic hybridization (CGH) data [5]. FISH and quantitative SNP analysis of tumor and matching normal cells and DNA from these patients revealed that these tumors constitutionally lacked paralog sequences but did not exhibit *DUSP22* deletions. Initial misinterpretation of *DUSP22* gene dosage was due to comparison to a commercial pool of normal DNAs, as usual in array CGH analysis [5], most of them carrying paralog alleles. Besides, Kadin, Feldman and colleagues reported 3 cases of cALCL with abnormal FISH patterns interpreted as bi-allelic *DUSP22* rearrangements [40]. Given the existence of the highly prevalent 16p11.2 paralog, the complex FISH profiles of such cases are hardly conclusive without concomitant analysis of matching normal cells and hybridization with 16p11.2 probes, as used here. Finally, two studies recently reported *DUSP22* 5′ region hypomethylation upon fire smoke exposure [41] or hypermethylation in Alzheimer's disease [42]. As we observed that the *DUSP22* gene 5′ region is not methylated in normal tissues, the methylation detected in these studies is likely to result from the presence of the paralog. Therefore, a comprehensive assessment of *DUSP22* status cannot be conducted without knowledge of its paralog copy number and 5′ methylation or deletion status.

Two out of the 20 studied cALCL cases harbored predicted inactivating somatic mutations, one of which also exhibiting monoallelic rearrangement and *DUSP22* silencing, while the second case had neither rearrangement nor deletion of the second allele. Most of our CTCL cases with monoallelic breakpoints thus did not exhibit a second hit that could explain silencing of *DUSP22*. A growing number of tumor suppressor genes are reported to be haploinsufficient, as heterozygous alterations leading to a 50% reduction in gene function are enough to promote oncogenesis [43]. Here, this hypothesis is however unlikely as complete silencing was always observed in tumors with monoallelic *DUSP22* rearrangements. As we also found that *DUSP22* is biallelically expressed in normal tissues, the second allele of 6p25.3 rearranged tumors is therefore somatically inactivated by an unknown transcriptional or post-transcriptional mechanism.

We then confirmed the existence of distinct *DUSP22* transcripts, produced by alternative splicing of exon 4 or intron 7 and predicting the production of 3 protein isoforms, only one of which being naturally detected in lymphoid T-cells. We showed that these *DUSP22* transcripts were all silenced in CTCL with 6p25.3 rearrangements. As anti-DUSP22 antibodies are not reliable for immuno-histochemical analyses [44], quantitative RT-PCR or RNA sequencing analyses are necessary to assess *DUSP22* expression levels.

Restoring expression of the physiological DUSP22 isoform in the 1301 DUSP22-deficient T cell leukemia cell line enhanced apoptosis and strongly impaired both clonogenicity in soft agar and tumorigenicity in immuno-deficient mice. Altogether, our study supports a tumor suppressor function of *DUSP22* and that its inactivation contributes to the development of PTCL. However, lack of spontaneous tumorigenesis in *Dusp22* knock-out mice [29] clearly indicates that *DUSP22* is not a gatekeeper tumor suppressor whose inactivation is sufficient to initiate tumor development. *DUSP22* rearrangements are moreover correlated with better clinical outcomes in systemic ALCL [14], but not in our series of cutaneous ALCL [11, 44] which however have an overall excellent prognosis. As for other tumor suppressors whose inactivation is a secondary event, loss of DUSP22 function may thus only contribute to transformation in cells already carrying other initiating oncogenic events. Global copy number analysis [5] did not reveal associations between *DUSP22* and other genomic alterations in our cALCL cases. *DUSP22* rearrangements are found mostly in ALK negative-ALCL [10, 11, 12, 14, 45], recently shown to harbor various molecular alterations leading to constitutive activation of the JAK/STAT3 pathway [46, 47]. Among other pathways [23, 24, 25, 26, 27, 28, 29], DUSP22 was previously shown to inhibit STAT3 signalling [26]. Mutations in this pathway may cooperate with *DUSP22* rearrangements in ALCL oncogenesis. Genome-wide massive parallel sequencing approaches will determine which molecular alterations coexist with DUSP22 inactivation in PTCL and may account for the favorable outcome of 6p25.3 rearranged tumors.

Besides, several types of cancers exhibit 6p25-pter allelic losses [48, 49, 50, 51, 52, 53, 54], suggesting the inactivation of a tumor suppressor gene in this region. Given its tumor suppressive behavior in PTCL, *DUSP22* is a candidate to be tested in tumors with 6p25 deletions, using a comprehensive approach such as described herein.

## MATERIALS AND METHODS

### Patients′ tissues and normal peripheral blood lymphocytes

Normal peripheral blood lymphocytes (PBL) and cutaneous T cell lymphomas (Supplementary Table S1) samples were collected and stored frozen at -140°C until nucleic acids and proteins extraction. Tumor samples presented at least 70% of cancer cells, based on histopathological review. Normal PBL were obtained upon Ficoll gradient centrifugation of blood samples from healthy donors at the établissement Français du Sang (Bordeaux, France). Other normal tissues (skin, lung, stomach, colon, kidney, bladder) were obtained from the Biobank of the University Hospital (Bordeaux, France). The study was performed after obtaining informed written consent from patients and healthy donors according to the Declaration of Helsinki Principles and was approved by the medical ethical committee of Aquitaine, France.

### Cell culture

The HH cell line was purchased from the Deutsche Sammlung von Mikroorganismen und Zellkulturen (DSMZ, Braunschweig, Germany) and MAC cell lines [33] were a generous gift from Pr Marshall Kadin (Providence, USA). Other cell lines (My-La, FE-PD, HUT78, 1301; Supplementary Table S1) were obtained from sources previously described [34, 35, 36, 37, 38, 39]. HH and 1301 cell lines were grown in RPMI 1640 supplemented with 10% fetal bovine serum (Life Technologies). FE-PD cells were grown in IMDM supplemented with 20% fetal bovine serum (Life Technologies).

### Fluorescent in situ hybridization (FISH)

Samples preparation, hybridizations and 6p25.3 locus *FISH* analyses were carried out following standard protocols, as previously described [11]. Probes are listed in Figure 2 and Supplementary Figures S4, S5 and S7.

### DNA and RNA extraction

High-molecular-weight genomic DNA was extracted from fresh or fresh-frozen biopsy samples, normal peripheral blood lymphocytes or cell lines using a standard protocol with proteinase K digestion, phenol/chloroform extraction and ethanol precipitation, as previously described [34]. Total RNA was extracted using TRIzol Reagent (Invitrogen) and treated with DNAse I (Invitrogen) to avoid contamination with genomic DNA.

### Gene expression analysis

Gene expression levels were determined by reverse transcription/real-time quantitative PCR (qRT-PCR), as previously described [34], using the comparative MNE (Mean Normalized Expression) method [55]. The primers used for qRT-PCR analyses are listed in Supplementary Table S2. Melting curves, gel electrophoresis and sequencing analyses showed that these primers amplified only the specific fragments.

### SNP genotyping

The genotype and allelic expression status of *DUSP22* and its paralog was initially determined by genotyping of SNPs *rs11242812* and *rs1046656*, performed by Sanger sequencing of PCR products obtained from genomic DNA and complementary DNA (cDNA) of lymphocytes from healthy donors. Sequencing reactions were carried out on a 3130xl Genetic Analyzer (Applied Biosystems), using the Big Dye Terminator kit (Applied Biosystems). Further SNPs genotyping and allelic expression status analyses of *DUSP22* and its paralog, *IRF4* and *EXOC2* genes were performed by restriction enzyme digestion of PCR products obtained from genomic DNA and cDNA of lymphocytes, normal tissues or cell lines. PCR products encompassing SNPs *rs11242812* (*DUSP22*, exon 7), *rs1129085* (*DUSP22*, exon 8, coding region), *rs1046656* (*DUSP22*, exon 8, 3′ UTR), *rs231651*5 (*IRF4*, exon 11) and *rs11242914* (*EXOC2*, exon 28) were respectively digested by *Msp*I, *Msp*I, *Bss*SI, *Hpy*CH4V and *Hae*III restriction endonucleases. Alleles of each SNP, digested or not by the respective endonucleases, were evaluated after 4% low melting agarose electrophoresis.

Quantitative SNP (qSNP) analysis was performed to determine allele-specific copy numbers for *rs1046656* (alleles C and T). These analyses were performed on genomic DNA isolated from normal PBL, peripheral T-cell lymphomas (PTCL) cases and lymphoid T-cell lines. We first designed allele-specific primers to analyze the allelic status of this SNP by real-time quantitative PCR, as previously described [34]. Optimal PCR amplification conditions were 95°C for 3 minutes followed by 45 cycles of 95°C for 20 seconds and 62°C for 20 seconds. Quantification of each SNP allele was performed according to the MNE (Mean Normalized Expression) method [55], here adapted to DNA copy numbers. Data were normalized using *KLK3* (located on 19q13.41) as a control gene, but also *ALB* (4q13.3), *CDH1* (16q22.1) or *CECR1* (22q11.2) to ensure that there was no alteration of *KLK3* copy numbers. Data from tumor DNA were compared with DNA from matching normal tissue from the same patient. Data from cell line DNA, DNA from a series of normal PBL was used as a reference. To validate this real time PCR, we also developed a restriction fragment length polymorphism-PCR assay, performed with fluorescently labeled primers, with numbers of cycles adjusted to fall within the linear range of the amplification. PCR products encompassing SNP *rs1046656* were digested by the *Bss*SI restriction endonuclease and quantified using capillary electrophoresis. Allele-specific copy numbers, determined by quantifying the area under curve of each SNP allele's peak, were normalized using *KLK3* as a control gene. The primers used for amplification of genomic DNA and cDNA fragments for SNP genotyping by sequencing, restriction enzyme digestion and gel electrophoresis, quantitative real time PCR or capillary electrophoresis are listed in Supplementary Table S3. Amplicons specificity was verified by gel electrophoresis and sequencing analyses.

### DNA methylation analysis

Genomic DNA was subjected to sodium bisulfite treatment using the EpiTect Bisulfite Kit, according to the manufacturer's instructions (QIAGEN, Hilden, Germany). Methylation was then analyzed using real-time quantitative methylation-specific PCR (qMSP) and confirmed for selected representative samples using bisulfite genomic sequencing (BGS), using standard protocols [56]. As for gene expression and gene copy number determination, qMSP was quantified using the using the comparative MNE (Mean Normalized Expression) method [55]. Data were displayed as percentage of unmethylated and methylated alleles. The primers used for qMSP and BGS analyses were designed using the Primer Express v 1.0 software (Applied Biosystems) and are listed in Supplementary Table S4. Specificity was verified by melting curves, gel electrophoresis and sequencing analyses.

### DUSP22 mutation analysis

Genomic DNA from normal and tumor tissues were screened for the presence of mutations by quantitative PCR amplification followed by high resolution melting (HRM) analysis on a LC480 device (Roche Diagnostics), using the manufacturer's kit, as previously described [57]. Amplicons exhibiting a melting profile (± 3 Standard Deviations) or temperature (Tm) differing from control DNA were subjected to Sanger DNA sequencing, as described in the SNP genotyping section. The primers, encompassing the 8 *DUSP22* gene exons and splice sites, used for HRM analysis and Sanger sequencing are listed in Supplementary Table S5.

### Lentiviral vectors and transduction

To generate expression vectors, the coding sequences of *DUSP22* isoforms were amplified by polymerase chain reaction (PCR) using complementary DNA (cDNA) from normal lymphocytes as a template. The following primers were used: exon 1 forward primer (5′-*aaactgcag*AGCCACCATGGGGAATGGGAT-3′, low capitals italic characters being an overhang and engineered *Pst*I site) with either intron 7 reverse primer (5′-*aaaacgcgt*TTAGGTCTCCGTCGTATAATTATCG-3′, low capitals italic characters being an overhang and engineered *Mlu*I site) or exon 8 reverse primer (*aaaacgcgt*CATTACAGTCTTCTGAGAAAGG, low capitals italic characters being an overhang and engineered *Mlu*I site). PCR products were cloned in a bicistronic self-inactivating (SIN) lentiviral vector, under the control of the myeloproliferative sarcoma virus enhancer, upstream of an internal ribosomal entry site (IRES2) followed by the ZsGreen fluorescent protein coding sequence. A similar lentiviral vector containing only the ZsGreen coding sequence under the control of the myeloproliferative sarcoma virus enhancer was used as a control. Constructs were verified by sequencing. Lentiviral vectors were produced by triple-transient transfection of 293T cells, the viral titer was determined and cell lines were transduced at an optimal multiplicity of infection (MOI) of 30 viral particles per cell, as previously described [58].

### Western blot analysis

DUSP22 protein levels were assessed by Western blot, following standard protocols. The antibodies used were DUSP22 (HPA031394, Sigma-Aldrich, St. St. Louis, MO 63103, USA, 16514-1-AP, Proteintech, Chicago, IL 60612, USA, and sc-47935, Santa Cruz Biotechnology, Dallas, TX 75220, USA) and α-Tubulin (Sigma-Aldrich).

### Flow cytometry, cell sorting, cell proliferation and viability assays

#### Cell sorting

The lentiviral infection efficiency of the tested cells was monitored by flow cytometry evaluating ZsGreen expression on a FACS Calibur and BD FACS Canto II (Becton Dickinson, San Jose, CA). Transduced 1301 and FE-PD cells were stained with DAPI (1μg/ml) for 15 min. Viable (DAPI negative)-transduced (ZsGreen positive) cells were sorted using a BD ARIA II device (BD Biosciences). Cell fraction purity was checked and selected cell populations were used for qRT-PCR, western blotting and phenotypic assays. Cell proliferation was evaluated by FACS at different time points (0, 3 and 6 days after plating) after staining with Cell trace™ Violet, according to manufacturer recommendations (Molecular Probes®, Eugene, OR, USA). Apoptosis/necrosis was evaluated by FACS after staining with an anti-annexin V-PE conjugated antibody according to manufacturer recommendations (BD Biosciences, Le Pont-de Claix, France) and Hoechst 33342 was added 5 minutes before sample acquisition. Acquisition and analysis were performed using a BD FACS Canto II (BD Biosciences) and the FlowJo software (Tree Star Inc.) respectively.

### Soft agar assays and tumorigenicity in immunodeficient mice

Clonogenicity and tumorigenicity assays with cells infected with control or DUSP22 ectopic expression vectors were performed as described [34].

### Statistical analysis

The statistical significance of differences in *DUSP22* expression levels and proliferation, viability, clonogenicity, or tumorigenicity of cells infected with control and *DUSP22* expression vectors was evaluated by the Student's t-test, using the MedCalc software. All graphs represent mean values ± s.e.m. *P* values are indicated on the figures, P ≤ 0.05 being considered significant.

## SUPPLEMENTARY MATERIALS






