# Supplementary material for: Molecular alterations and tumor suppressive function of the DUSP22 (Dual Specificity Phosphatase 22) gene in peripheral T-cell lymphoma subtypes
Source: Oncotarget. 2016 Sep 10;7(42):68734–48. doi: 10.18632/oncotarget.11930 (PMC5356586; doi:10.18632/oncotarget.11930)
Supplement: Supplementary file 1 [file oncotarget-07-68734-s001.pdf]

Molecular alterations and tumor suppressive function of the *DUSP22 (Dual Specificity Phosphatase 22)* gene in peripheral T-cell lymphoma subtypes

SUPPLEMENTARY MATERIAL

Molecular characterization of the paralog of the *DUSP22* gene

The occurrence of hybridization signals on both 6p25.3 and 16p11.2 loci with probes encompassing *DUSP22* (Supplementary Figure S4, Figure 2A) was consistent with bioinformatics analyses predicting the existence of a closely related paralog of *DUSP22* on 16p11.2 [31, 32].

The high degree of sequence similarity predicted between *DUSP22* and its paralog would inevitably complicate the interpretation of cytogenetic and molecular analyses of the *DUSP22* gene. It was thus essential to determine the molecular features of the paralog in order to develop a comprehensive strategy to analyze unambiguously the status of *DUSP22*.

First, we developed a 3 color FISH approach, with a probe encompassing *DUSP22* (thus hybridizing also on the paralog when present) combined with 6p25.3 and 16p11.2 loci specific probes (Figure 2A, Supplementary Figure S5A). In agreement with bioinformatics [31, 32], this analysis revealed that the paralog was subjected to copy number variations (CNV), being absent in 10% of normal PBL and detected on one or both chromosomes 16 in respectively 30% and 60% of the cases (Figure 2A, Supplementary Figure S5A and Supplementary Table S6). Predictions of Genovese *et al.* about this paralog were based on heterozygosity frequency of single-nucleotide polymorphisms (SNPs) and haplotype segregation [31, 32].

We thus genotyped 3 of the 6 SNPs analyzed in this previous study [31] (Supplementary Figure S2) on PBL from unrelated healthy donors. We observed excess of heterozygosity (90% for *rs11242812*, 65%, for *rs1129085* and 90% for *rs1046656*), reminiscent of paralog sequences [31], and alleles co-segregation identified 3 haplotypes: G-G-C, G-A-C and A-G-T (Figure 2B, Table 1, Supplementary Figure S5B and Supplementary Table S6). In 10% of individuals, normal PBL showed only 6p25.3 *DUSP22*-specific FISH signals and carried exclusively the G-G-C and/or G-A-C haplotypes. Conversely, in 90% of individuals, the A-G-T haplotype was present and always associated with G-G-C and/or G-A-C haplotypes and with additional FISH signals on one or both 16p11.2 paralog alleles (Figure 2A, 2B, Table 1, Supplementary Figures S5A, S5B and Supplementary Table S6). We then analyzed the SNPs allelic expression status in normal PBL and various normal tissues. We found that the G-G-C and G-A-C alleles, which appear specific of *DUSP22*, were always transcribed in normal cells, while the A-G-T allele, which correlates with the presence of the 16p11.2 paralog, was silent or barely expressed in most carriers (Figure 2B, Table 1, Supplementary Figure S5C, Supplementary Figure S6A, S6C and Supplementary Table S6). Genovese *et al.* commented that the paralog was expressed similar to the reference *DUSP22* gene [31]. Actually, re-analysis of their RNA-sequencing reads data for 4 of the investigated SNPs, including *rs11242812* and *rs1046656* also evaluated herein, clearly showed that the paralog was mostly silent (Figure 5 in [31]). Only RNA-sequencing data of SNPs *rs3778605* and *rs1129085* could suggest expression of the paralog [31]. Our study of *rs1129085* suggested that transcripts from the G allele, shared between *DUSP22* and its paralog, could possibly have been misinterpreted as expression of the paralog in the report of Genovese *et al.* [31], as 78% of individuals carry at least one expressed G allele of *rs1129085* on *DUSP22* (Supplementary Table S6). Or this is due to misattribution of the A allele of *rs1129085* (which is specific of *DUSP22* and thus always expressed when present) to the paralog, as it can be understood from Supplementary Table S9 in [31].

Our data indicating that the paralog was transcriptionally inactive or hypomorphic prompted us to examine the epigenetic status of *DUSP22* and its paralog. We found that partial methylation of a 5'-CpG island was frequent in normal cells, including PBL (Figure 2C, 2D, Supplementary Figures S5D and S6B, S6D). Matching with FISH and SNP data revealed that cases without 16p11.2 paralog sequence showed no CpG island methylation, while methylation was detected in most carriers of the paralog and proportional to its copy numbers (Figure 2, Supplementary Figures S5C, S5D and S6B, S6D and Supplementary Table S6). In normal cells, methylation was thus found on paralog but not *DUSP22* sequences (Table 1). Nonetheless, rare cases with 16p11.2 paralog sequence detected by FISH and silent A-G-T haplotype lacked CpG island methylation (*e.g.* PBL13, Figure 2B, 2D and Supplementary Table S6). Such cases likely harbor the paralog allele with a predicted 5' deletion (Supplementary Figure 9 in [31]), as indicated by quantitative PCR (Supplementary Figures S5C and S5D). As this deletion encompasses promoter and exon 1 sequences, this would account for lack of paralog 5' methylation.

Similar methylation and allelic expression patterns were observed in all other normal tissues tested (Supplementary Table S6 and Supplementary Figures S6C and S6D). The paralog is thus either methylated or deleted in its 5' region and transcriptionally inactive in most instances (Figure 2B, 2D, Table 1, Supplementary Figures

S5, S6 and Supplementary Table S6). However, in rare samples (4/47, 10.6%), transcripts from the A-G-T allele were fully expressed as well (*e.g.* PBL12 in Figure 2B, Supplementary Table S6). Our hypothesis is that in such individuals an active A-G-T haplotype may be present on the 6p25.3 locus and correspond to a rare ancestral allele of *DUSP22* from which the paralog was duplicated (Supplementary Figure S6E).

In addition to expression data, FISH, methylation and SNP analyses showed that *IRF4* and *EXOC2* have no paralog and do not exhibit significant alteration in the tested cutaneous T-cell lymphomas (Supplementary Figure S3).

SUPPLEMENTARY FIGURES AND TABLES

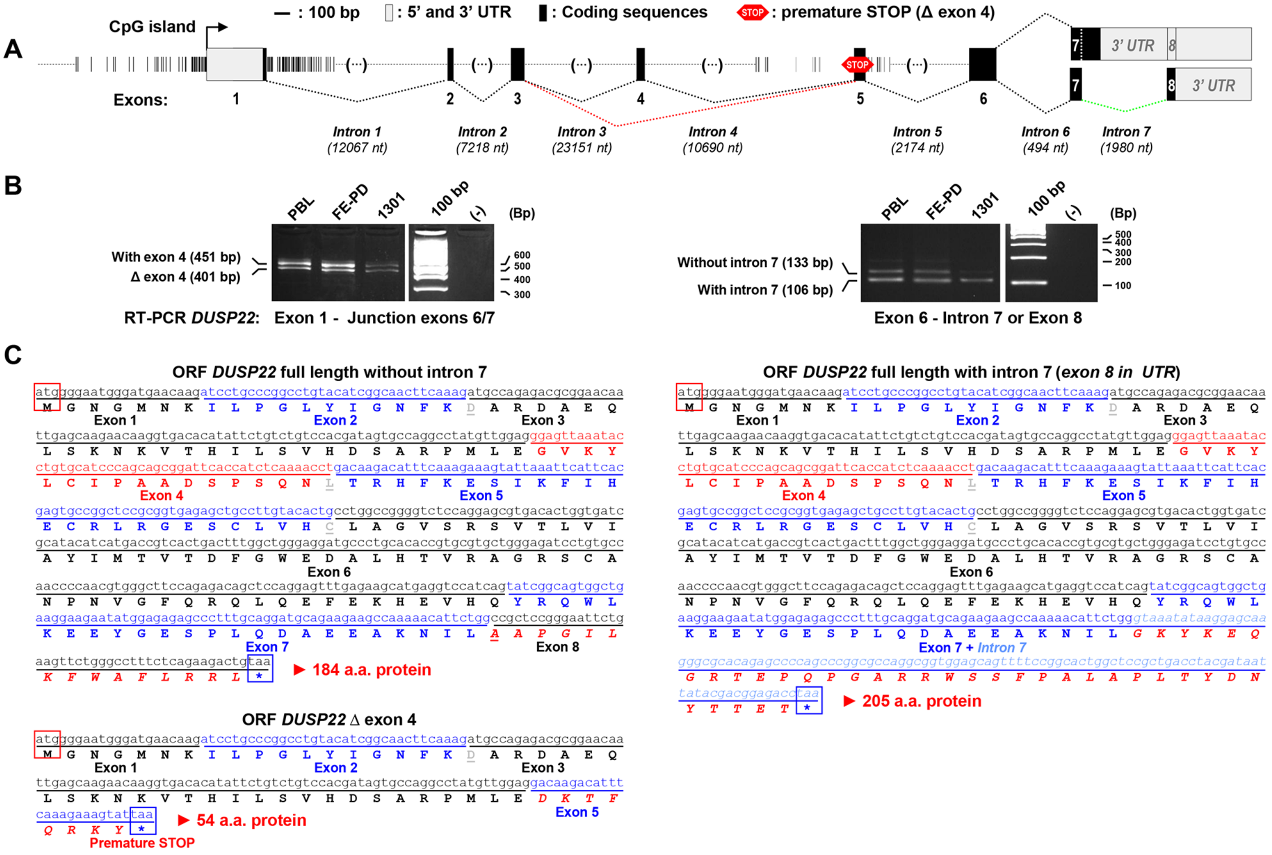

Supplementary Figure S1: Description of DUSP22 isoforms. A. Structure of the human *DUSP22* gene and alternative transcripts. Numbered boxes indicate the exons, with 5' and 3' untranslated (UTR) regions in grey and coding region in black. Nt: nucleotides. The black arrow indicates the position of the transcription initiation site. Black and red dotted lines indicate the alternative splicings, concerning exon 4 and intron 7. Four alternatively spliced transcripts have been identified: 1) transcripts containing 8 exons with all introns spliced; 2) transcripts with the same 8 exons but retaining intron 7 (exon 8 then being in 3' UTR); 3 and 4) the above-mentioned transcripts but with exon 4 spliced out (Δ exon 4 transcripts). The red box highlights the presence of a premature STOP codon which is in frame in Δ exon 4 transcripts. **B.** RT-PCR analyses of normal lymphocytes (PBL) and different lymphoid (1301, FE-PD) cell lines illustrating the existence of *DUSP22* alternative transcripts. Left panel: RT-PCR was performed with a forward primer within exon 1 and a reverse primer at the junction between exons 6 and 7, revealing the presence of the expected fragment as well as a 50 base pairs shorter fragment which, by Sanger sequencing, was shown to exhibit exon 4 splicing (Δ exon 4 transcripts). Right panel: RT-PCR was performed with a forward primer within exon 6 and two reverse primers: one within the exon 8 coding sequence and one within the part of intron 7 which is present only in transcripts which were shown, by Sanger sequencing, to retain intron 7 (Sequence framed in red in Supplementary Figure S2). This analysis revealed that transcripts with and without intron 7 splicing are present in all tested samples, in variable proportions depending on the cells. **C.** Complete complementary DNA (cDNA) and peptidic sequence of DUSP22 isoforms. Based on their cDNA sequences and the positions of possible STOP codons (see also Supplementary Figure S2), the 4 different types of transcripts indicated in (**A**) are predicted to encode 3 distinct protein isoforms: transcripts containing all 8 exons and showing intron 7 splicing are predicted to encode a 184 amino-acids protein (Top left panel); 2) transcripts containing all 8 exons and retaining intron 7 (exon 8 then being in 3' UTR) are predicted to encode a 205 amino-acids protein (right panel); the two previous types of transcripts with exon 4 spliced out (Δ exon 4 transcripts) are predicted to encode a 54 amino-acids protein (Bottom left panel). Nucleotidic sequences of exons are indicated alternatively in black and blue and underlined, in order to highlight the limits of each exon. Amino-acids corresponding to each codon are indicated bellow the cDNA sequences. Amino-acid residues encoded by codons overlapping two exons are marked in grey. The sequence of the alternatively spliced exon 4 and corresponding amino-acids are indicated in red. The part of exon 7 which is present and coding only in transcripts retaining intron 7, as well as the sequence of exon 8 which is coding only in transcripts with intron 7 splicing are indicated in italic. The ATG initiating codon is framed in red and the STOP codon for each isoform is framed in blue. Isoform-specific amino-acids are in italic and red. These 3 protein isoforms differ in their C-terminal domains.

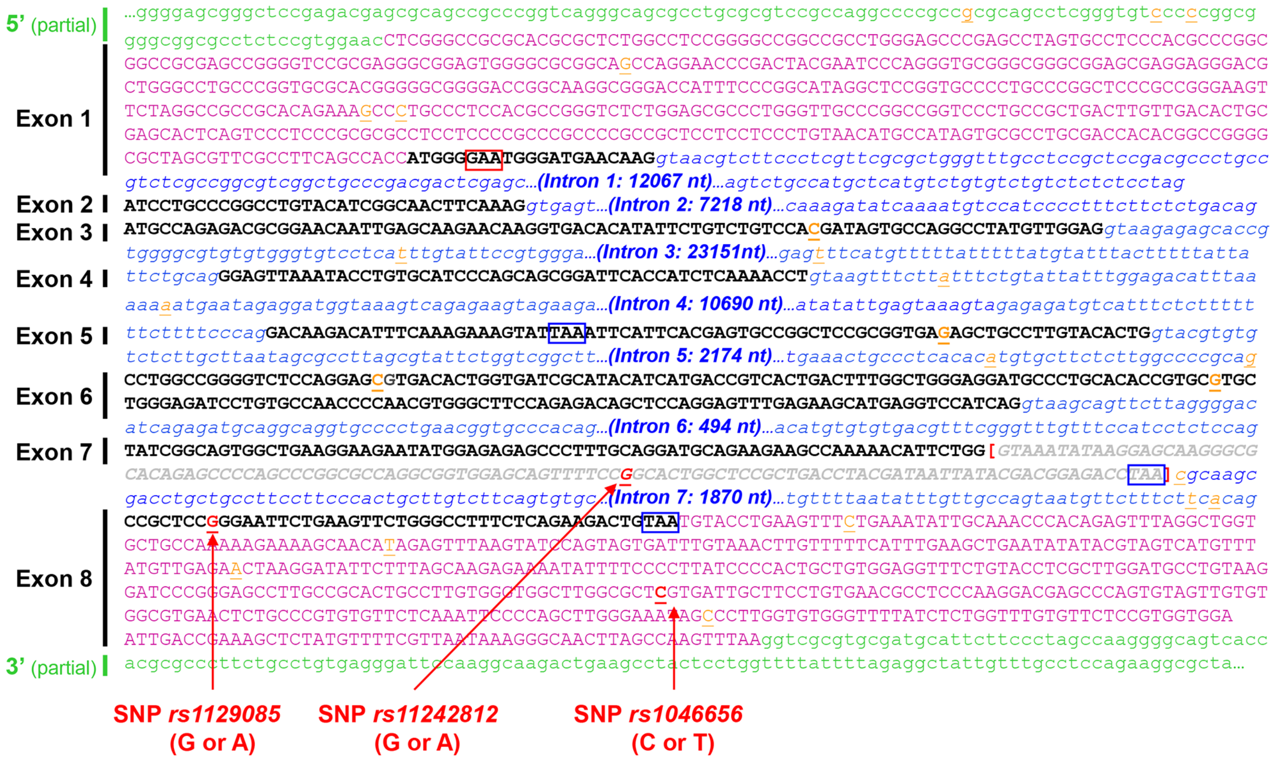

Supplementary Figure S2: Partial sequence of the *DUSP22* gene. Full coding sequence and partial untranslated (UTR) and intron sequences are shown. 5' and 3' UTR regions are in purple characters and the 5' and 3' flanking regions are in green. The coding region is in bold black characters. The sequence in grey italic capitals delineated by red brackets is present and coding only in mRNA from the isoform lacking intron 7 splicing (Supplementary Figure S1). The exon 8 sequence in bold black is coding only in the transcripts with intron 7 splicing (Supplementary Figure S1). The ATG translation initiation codon, common to all isoforms (Supplementary Figure S1), is framed in red. The 3 possible STOP codons, depending on the isoform, are framed in blue. The potential STOP highlighted within exon 5 is in frame only in the Δ exon 4 splice variant (Supplementary Figure S1). The potential STOP highlighted within intron 7 is present only in the splice variant retaining intron 7 (Supplementary Figure S1). The potential STOP highlighted within exon 8 is in frame only in the splice variant without intron 7 and retaining all exons (Supplementary Figure S1). Intron sequences are in blue characters. The reported single nucleotide polymorphisms (SNPs) appear in orange and underlined and the 3 SNPs investigated in this study *rs11242812* (G or A), *rs1129085* (G or A) and *rs1046656* (C or T) are in red and underlined, and indicated by red arrows.

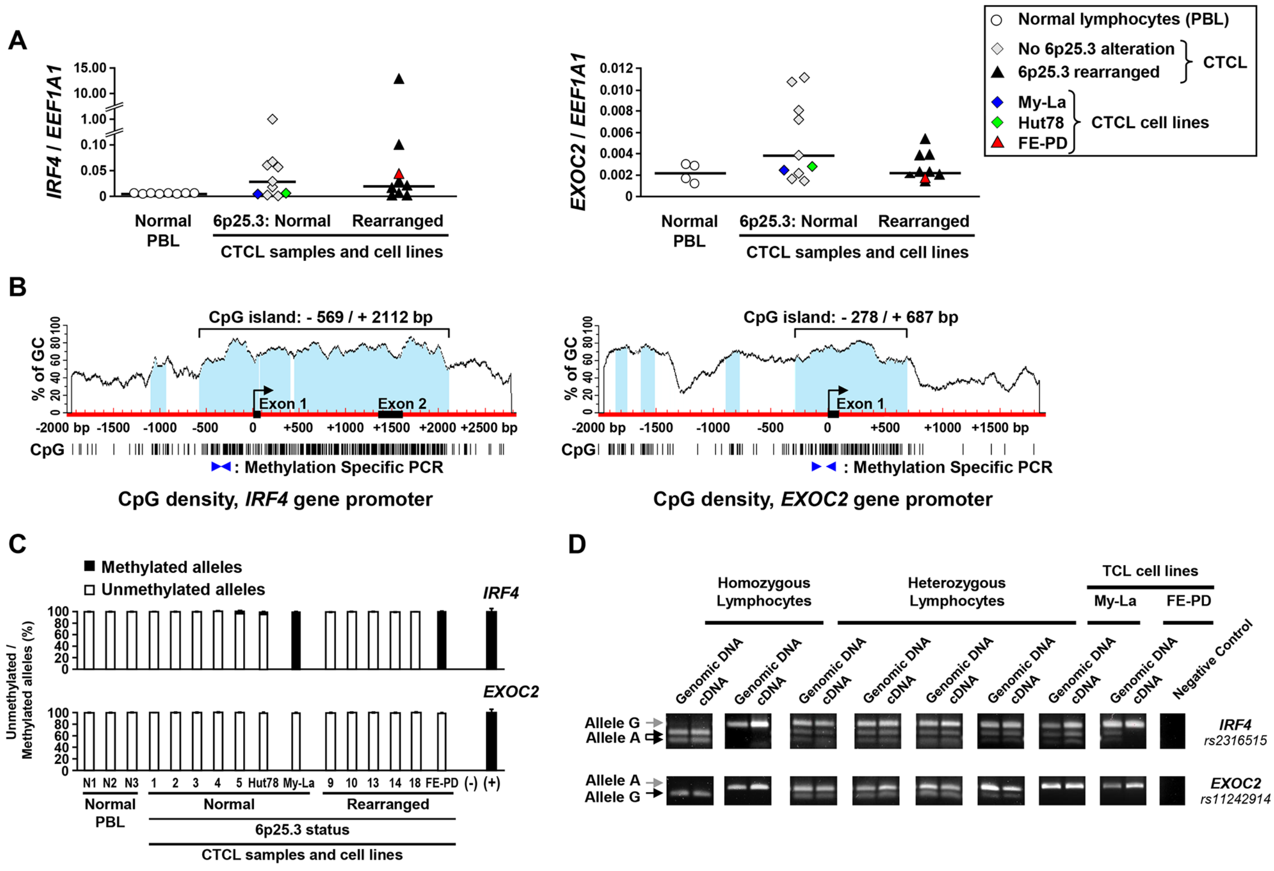

Supplementary Figure S3: Expression levels, methylation and allelic expression status of the *IRF4* and *EXOC2* genes in normal lymphocytes and cutaneous T-cell lymphomas. A. Quantitative real-time reverse transcription-PCR (qRT-PCR) analysis of *IRF4* (Left panel) and *EXOC2* (Right panel) transcript levels on RNA isolated from normal peripheral blood leukocytes (PBL) and cutaneous T-cell lymphomas (CTCL) tumors and cell lines with and without 6p25.3 rearrangements. *IRF4* and *EXOC2* transcript levels were normalized for *EEF1A1* gene expression. CTCL exhibit occasional overexpression of *IRF4* or *EXOC2* without correlation with 6p25.3 genomic status, indicating that these genes are not the targets of 6p25.3 rearrangements in ALCL. **B.** Schematic representation of the 5' region of the *IRF4* and *EXOC2* genes. Exons are indicated by black boxes, the transcription start sites by a curved arrow and the primers used for methylation specific PCR (MSP) analyses by blue arrowheads. Both *IRF4* and *EXOC2* genes harbor large CpG islands (vertical bars indicate CpG sites), spanning from the promoter through the beginning of intron 2 for *IRF4* (Left panel) and encompassing the promoter, exon 1, and beginning of intron 1 for *EXOC2* (Right panel). **C.** Quantitative methylation specific PCR (qMSP) analysis of *IRF4* (Top panel) and *EXOC2* (Bottom panel) in normal lymphocytes (PBL) and cutaneous T cell lymphoma (CTCL) samples with and without 6p25.3 rearrangements. No DNA (-) and *in vitro* methylated DNA (+) were used as negative and positive controls. Relative proportions (%) of unmethylated and methylated alleles (Mean ± SEM from independent measurements) are shown. Excepted the My-La and FE-PD cell lines, both showing strong *IRF4* 5' CpG island methylation, none of the tested normal or tumor samples exhibited any significant 5' CpG island methylation of either *IRF4* or *EXOC2* genes. **D.** Representative examples of genotype and allelic expression status analysis of two SNPs (*rs2316515* -A or G- and *rs11242914* -G or A-), respectively located within exon 11 (3' UTR) of *IRF4* and exon 28 (3' UTR) of *EXOC2*. Fragments encompassing these SNPs were amplified by PCR from genomic DNA or complementary DNA (cDNA) from normal PBL. PCR products encompassing SNPs *rs2316515* and *rs11242914* were respectively digested by *Hpy*CH4V and *Hae*III restriction endonucleases and analyzed by 4% low melting agarose electrophoresis. Arrows indicate each SNP allele (digested or undigested). Electrophoresis profiles show that normal lymphocytes heterozygous for the SNPs, based on restriction polymorphism analysis of genomic DNA, exhibit bi-allelic expression of *IRF4* and *EXOC2* genes (detection of transcripts from both alleles upon cDNA analysis). These data demonstrate the absence of allelic inactivation of these genes in normal lymphocytes, in agreement with the absence of CpG island methylation. Concerning the My-La and FE-PD cell lines, both showing strong *IRF4* 5' CpG island methylation, only FE-PD exhibited absence of expression of one allele (allele A) of *IRF4*, although present at the DNA level. The fact that both *IRF4* alleles were expressed in My-La cells and that one allele was expressed in FE-PD cells, despite complete *IRF4* 5' CpG island methylation, suggests that there might be alternative *IRF4* transcription start sites insensitive to hypermethylation of this region. All tested *EXOC2* SNPs were not informative in both cell lines (homozygous at the DNA level), preventing allelic expression status analysis of this gene in these cells.

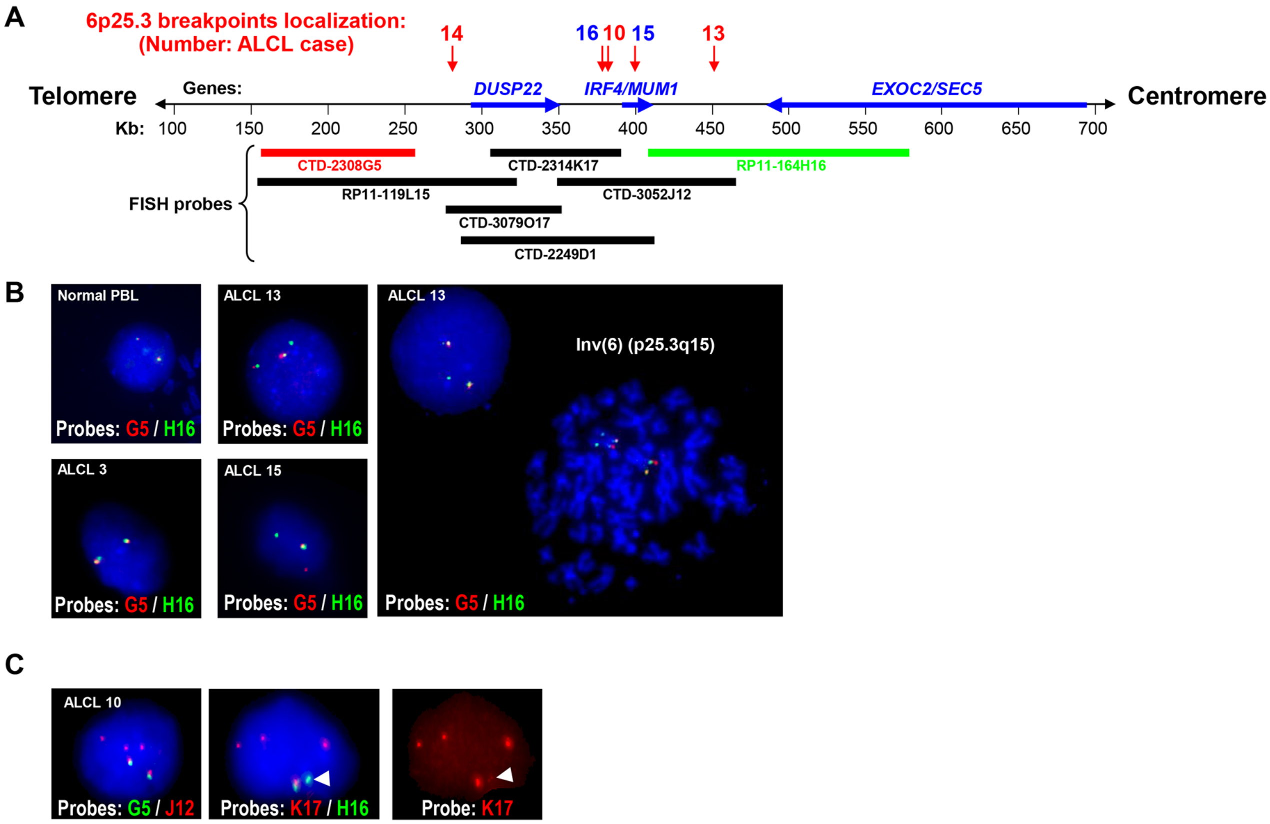

Supplementary Figure S4: Localization of 6p25.3 breakpoints in peripheral T cell lymphomas. A. Schematic representation of the 6p25.3 chromosomal band, with position of the genes and probes used for fluorescent in situ hybridization (*FISH*) to map the localization of rearrangements breakpoints (red arrows) in cutaneous anaplastic large cell lymphomas (ALCL) samples. BAC probes indicated in black were used to specify the position of the 6p25.3 breakpoint in some cases **B.** Interphase *FISH* analysis of normal peripheral blood lymphocytes (PBL) and cutaneous ALCL illustrating the presence of rearrangements in case 13 (breakpoint within the region covered by probe RP11-164H16 -indicated H16 on the Figure-) and case 15 (breakpoint between probes CTD-2308G5 -indicated G5 on the Figure- and RP11-164H16). DAPI (4′,6-diamidino-2-phenylindole dihydrochloride), visualized in blue, was used to stain nuclear DNA and chromosomes. For ALCL case 13, metaphases could also be analyzed, indicating the presence of a chromosomal inversion -Inv(6)(p25.3q15)-, consistent with karyotype data previously published by our group [9]. **C.** Illustration of breakpoint mapping in ALCL case 10 with different FISH probes (indicated on each picture). Probes CTD-3052J12 -indicated J12 on the Figure- and CTD-2314K17 -indicated K17 on the Figure-cross-hybridized to another locus, later identified as a *DUSP22* paralog on 16p11.2 (Figure 2A, Supplementary Figures S5A, S5B, S6E and [31, 32]).

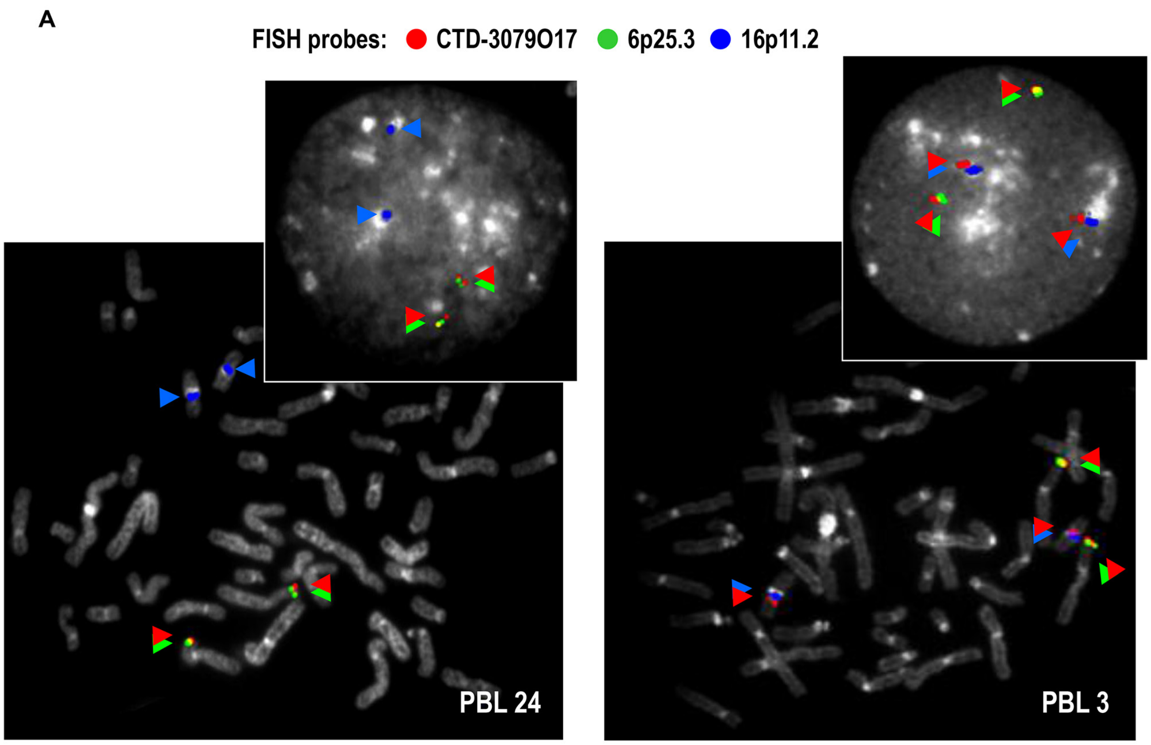

Supplementary Figure S5: Combined fluorescence *in situ* hybridization and molecular analytical strategy to determine *DUSP22* and 16p11.2 paralog copy numbers and allelic and methylation status. A. Metaphase and interphase *FISH* analysis of normal peripheral blood lymphocytes (PBL) with a 6p25.3-specific probe (RP11-164H16, Spectrum Green labeled), a probe encompassing the *DUSP22* gene (CTD-3079O17, Spectrum Red labeled), and a 16p11.2-specific probe (RP11-488I20, Spectrum Gold labeled, here displayed in blue (pseudocolor) as colocalization of signals from the former probes may appear in yellow). DAPI (4′,6-diamidino-2-phenylindole dihydrochloride), here visualized in grey (pseudocolor), was used to stain nuclear DNA and chromosomes. (*Continued*)

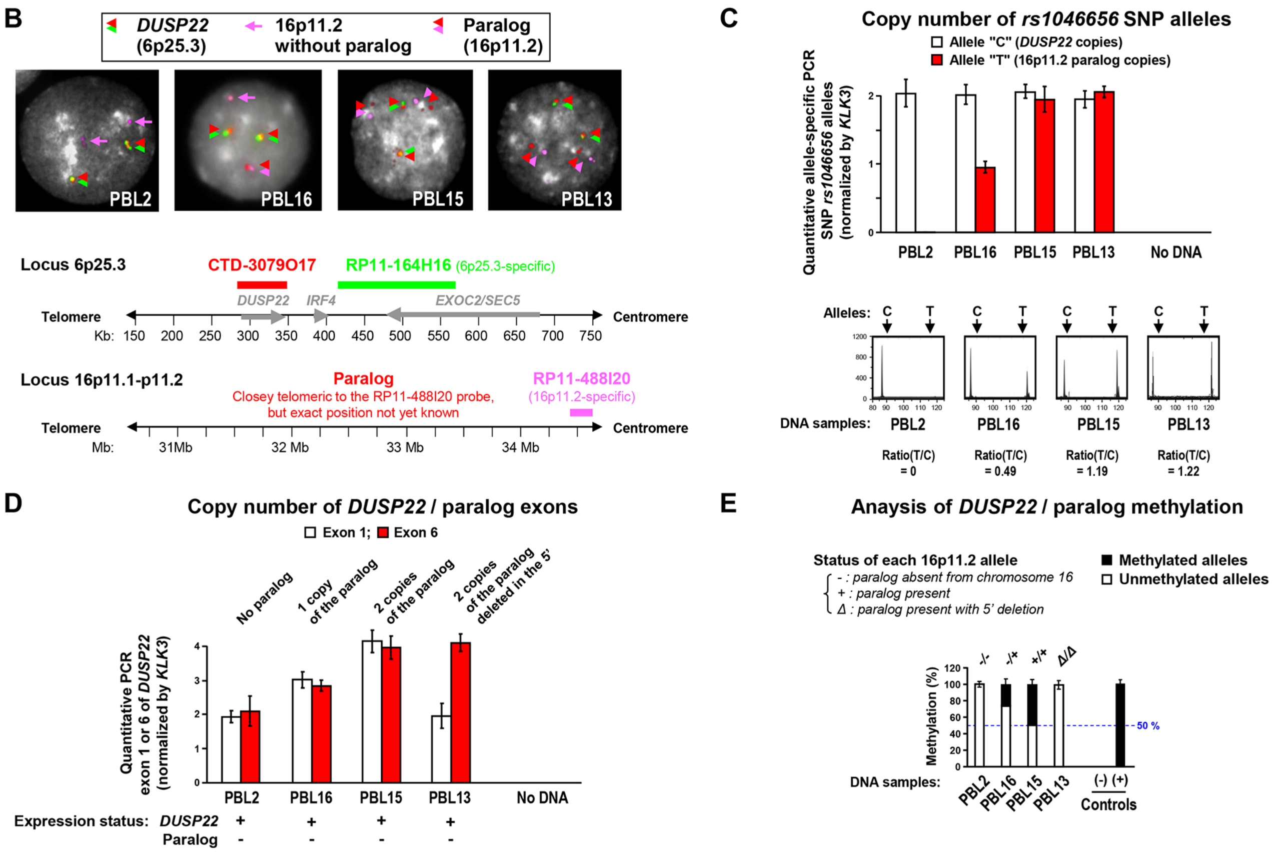

Supplementary Figure S5: (*Continued*) Combined fluorescence *in situ* hybridization and molecular analytical strategy to determine *DUSP22* and 16p11.2 paralog copy numbers and allelic and methylation status. B. Interphase fluorescence in situ hybridization (*FISH*) analysis of normal peripheral blood lymphocytes (PBL) with a 6p25.3-specific probe (RP11-164H16, Spectrum Green labeled), a probe encompassing the *DUSP22* gene (CTD-3079O17, Spectrum Red labeled), and a 16p11.2-specific probe (RP11-488I20, Spectrum Gold labeled, here displayed in pink (pseudocolor) as colocalization of signals from the former probes may appear in yellow). DAPI (4′,6-diamidino-2-phenylindole dihydrochloride), here visualized in grey (pseudocolor), was used to stain nuclear DNA. On panels (A) and (B), cases exhibiting, in addition to 6p25.3, one or two additional CTD-3079O17 probe signals, illustrate the predicted existence of a *DUSP22*-paralog subjected to copy number variations at the 16p11.2 locus [31, 32] (See also Figure 2A). This paralog was absent from PBL2 lymphocytes and present in 1 copy in PBL25 and 2 copies in PBL13 and PBL15. **C.** Top panel: Quantitative real-time PCR analysis of *rs1046656* SNP allele-specific copy numbers in normal PBL. Data were normalized using *KLK3* copy numbers. Mean ± SEM from independent measurements are shown. Bottom panel: Capillary electrophoresis-based analysis of SNP *rs1046656* alleles copy numbers in normal PBL. PCR was performed to fall within the linear range of the amplification, with fluorescently labeled primers. PCR products were digested by the *Bss*SI restriction endonuclease and analyzed by capillary electrophoresis. Area under curve was quantified each SNP allele's peak and normalized using *KLK3*. Allelic ratios are indicated bellow each electrophoregram. These allele specific analyses (confirmed in larger series of normal samples, see Supplementary Table S6) indicated that detection of the T allele of SNP *rs1046656* correlated with the presence and copy numbers of the 16p11.2 paralog (absent in PBL2, 1 copy in PBL25 and 2 copies in PBL13 and PBL15 lymphocytes). **D.** Quantitative real-time PCR analysis of *DUSP22* exons 1 and 6 in normal PBL. Data were normalized using *KLK3* copy numbers. Mean ± SEM from independent measurements are shown. The expression status of *DUSP22* (allele C of SNP *rs1046656*) and its paralog (allele T of SNP *rs1046656*) (see text and Supplementary Table S6) is indicated bellow the graph. Enhanced copy numbers (from 2 to 3 or 4 copies) correlated with the presence of 1 copy (in PBL25) or 2 copies (PBL13 and PBL15) of the 16p11.2 paralog. The difference between exon 6 and exon 1 copy numbers (PBL13) is consistent with the existence of paralog alleles deleted in their 5' region, including exon 1 and the 5' CpG island, as predicted by analysis of next generation sequencing reads numbers (Supplementary Figure 9 in Ref. [31]). **E.** Quantitative methylation specific PCR (qMSP) analysis of *DUSP22*/16p11.2 paralog 5' CpG island in normal PBL. No DNA (-) and *in vitro* methylated DNA (+) were used as negative and positive controls. Relative proportions (%) of unmethylated and methylated alleles (Mean ± SEM from independent measurements) are shown. Detection of methylation with the presence and copy numbers of the 16p11.2 paralog (absent in PBL2, and representing ≈33% in PBL25 -1 paralog copy- and ≈50% in PBL15 -2 paralog copies-), except in PBL13 for which FISH and molecular analysis (see Supplementary Figures S5A-S5D) was consistent with the presence of 2 paralog alleles deleted in the 5' region, such deletion encompassing the region methylated in other paralog alleles.

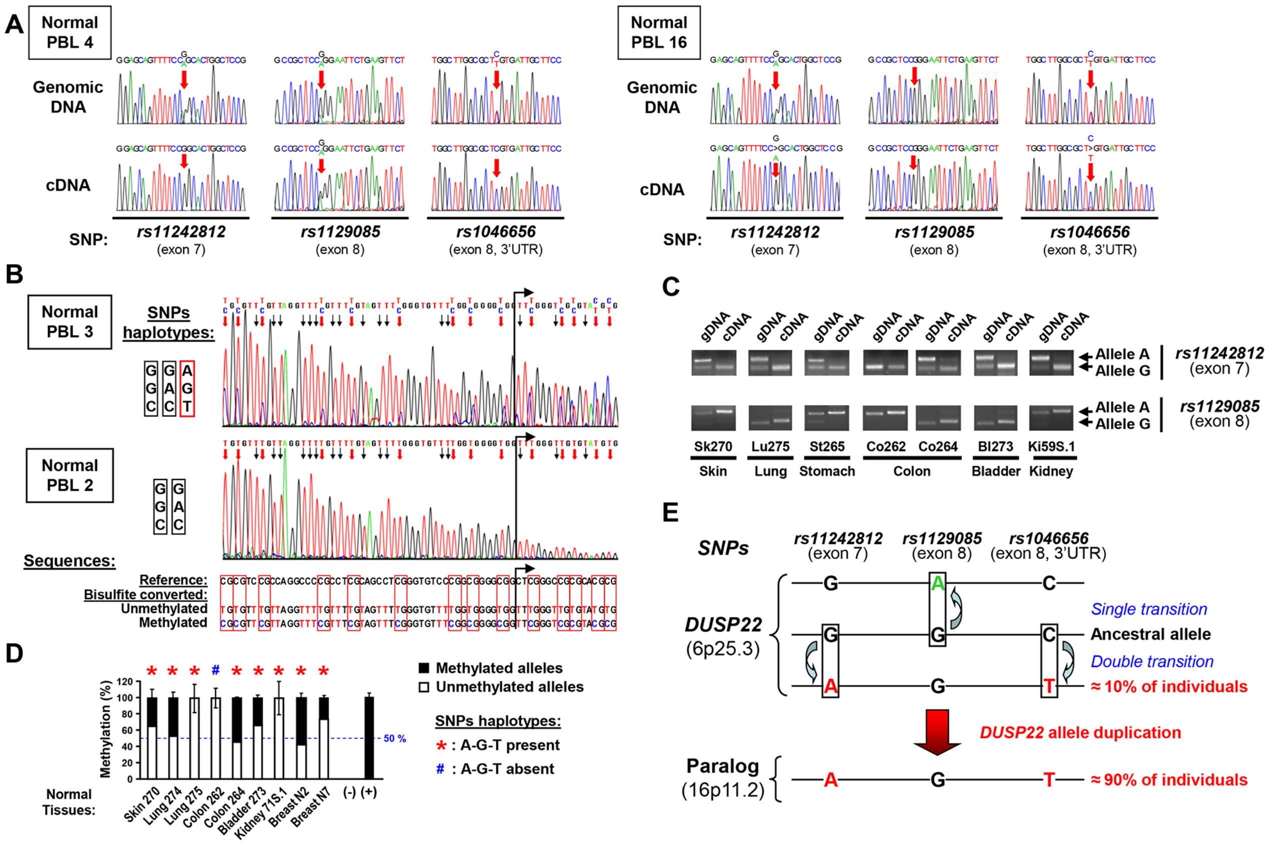

Supplementary Figure S6: Allelic expression status and methylation of the *DUSP22* gene in normal tissues. A. Examples of genotype and allelic expression status of SNPs *rs11242812* (G or A), *rs1129085* (G or A) and *rs1046656* (C or T), located within exons 7 and 8 of *DUSP22*, determined by Sanger sequencing of PCR products obtained from genomic DNA (gDNA) and complementary DNA (cDNA) of peripheral blood lymphocytes (PBL) from healthy donors. **B.** High-resolution methylation analysis of individual CpG sites around the *DUSP22* transcriptional start site in representative examples of normal lymphocytes with distinct genotypes for SNPs *rs11242812*, *rs1129085* and *rs1046656*, as indicated. Black arrows indicate the positions of non-CpG Cytosines that are not methylated and systematically converted to Thymines (see reference and bisulfite converted sequences as well as Sanger sequencing profiles). Red arrows indicate the positions of CpG-associated Cytosines which are susceptible to methylation and therefore converted or not to Thymine depending on their methylation status (see reference and bisulfite converted sequences as well as Sanger sequencing profiles). Red boxes on the sequences highlight the positions of the CpG sites and the transcription start site is indicated by the curved arrow. **C.** Representative examples of genotyping (gDNA) and allelic expression (cDNA) of SNPs *rs11242812* and *rs1129085* in normal tissues, determined by PCR and restriction enzyme (*Msp*I) digestion. **D.** Quantitative methylation specific PCR (qMSP) analysis of *DUSP22*'s 5' CpG island in DNA from different normal tissues with distinct SNPs genotypes, as indicated. No DNA (-) and *in vitro* methylated DNA (+) were used as negative and positive controls. Percentages of unmethylated and methylated alleles (Mean ± SEM from independent measurements) are shown. **E.** Representation of the alleles of *DUSP22* and its paralog. Based on allelic segregation and expression status, we identified 3 haplotypes of SNPs *rs11242812*, *rs1129085* and *rs1046656*: G-G-C and G-A-C haplotypes are specific of *DUSP22*, while the A-G-T haplotype may correspond to a rare *DUSP22* allele from which the inactive paralog was duplicated (see text and Supplementary Table S6).

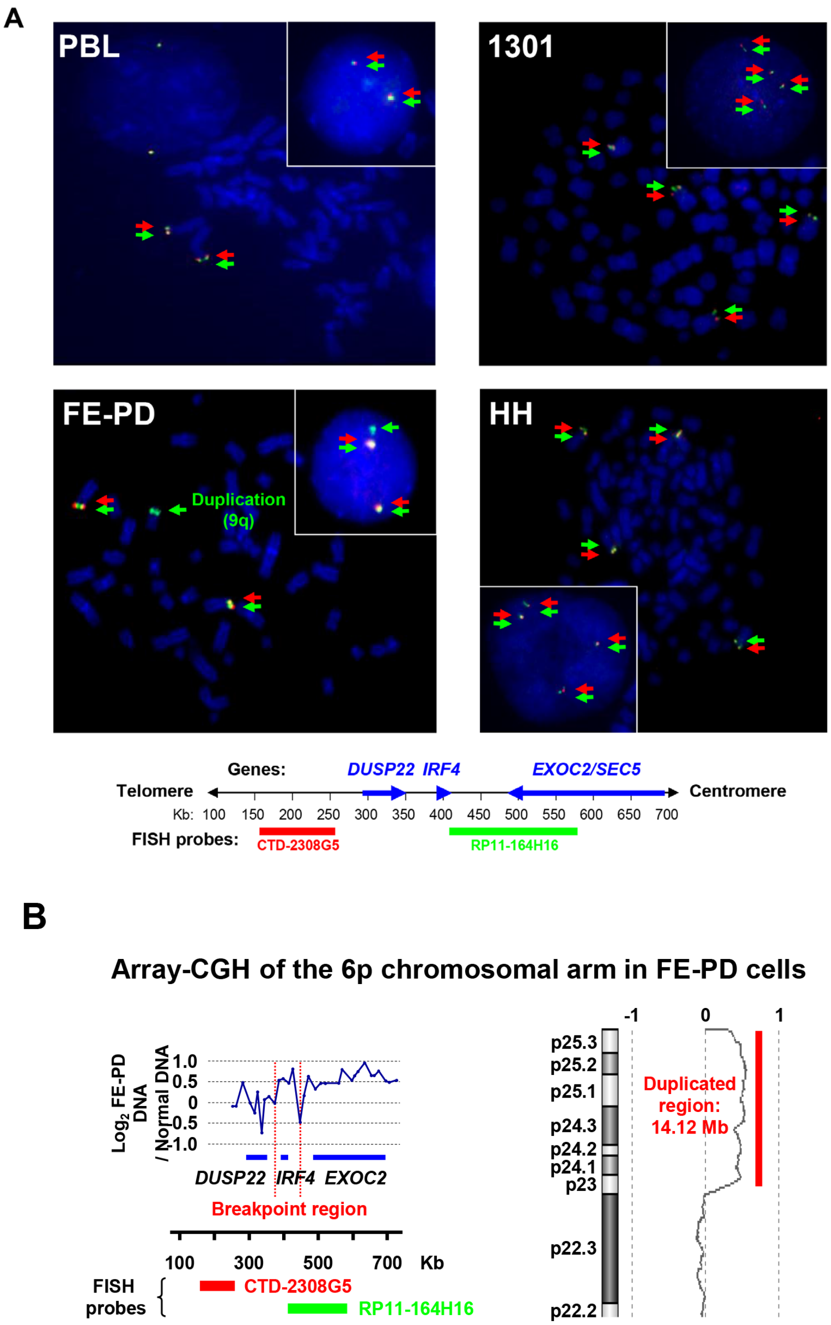

Supplementary Figure S7: Status of the 6p25.3 locus in lymphoid T-cell lines. A. Metaphase and interphase (inserts) fluorescent in situ hybridization (*FISH*) analysis of normal peripheral blood lymphocytes (PBL) and lymphoid T-cell lines and schematic representation of the 6p25.3 locus, with position of the genes and probes (CTD-2308G5 -labeled in red- and RP11-164H16 -labeled in green) used for *FISH*. DAPI (4′,6-diamidino-2-phenylindole dihydrochloride), visualized in blue, was used to stain nuclear DNA and chromosomes. FE-PD cells exhibit 2 copies of the 6p25.3 locus without evidence of rearrangement, as illustrated by the presence of colocalization of hybridization signals from the 2 probes flanking the *DUSP22* and *IRF4* genes. However, as illustrated by the presence of an additional hybridization signal with the RP11-164H16 probe, FE-PD cells harbor a complex rearrangement leading to duplication of a region located downstream of *DUSP22* on a locus identified by DAPI banding and multicolor FISH as 9qter (data not shown). The breakpoint of this rearrangement was mapped between *DUSP22* and *IRF4* (see bellow, Supplementary Figure S7B). 1301 and HH cells, both harboring a nearly tetraploid karyotype, exhibit in most cells 4 copies of the 6p25.3 locus, without evidence of rearrangement. **B.** Detailed 6p25.3 (left panel, with position of the genes and the FISH probes used in **A**) and global 6p22.2-pter (right panel) view of oligonucleotide microarray-based comparative genomic hybridization (array-CGH) of FE-PD cells DNA (FE-PD DNA/control DNA ratios are shown). This microarray analysis, performed as previously reported by our group [5], showed duplication of a 14.12 Mb region located downstream of *DUSP22*.

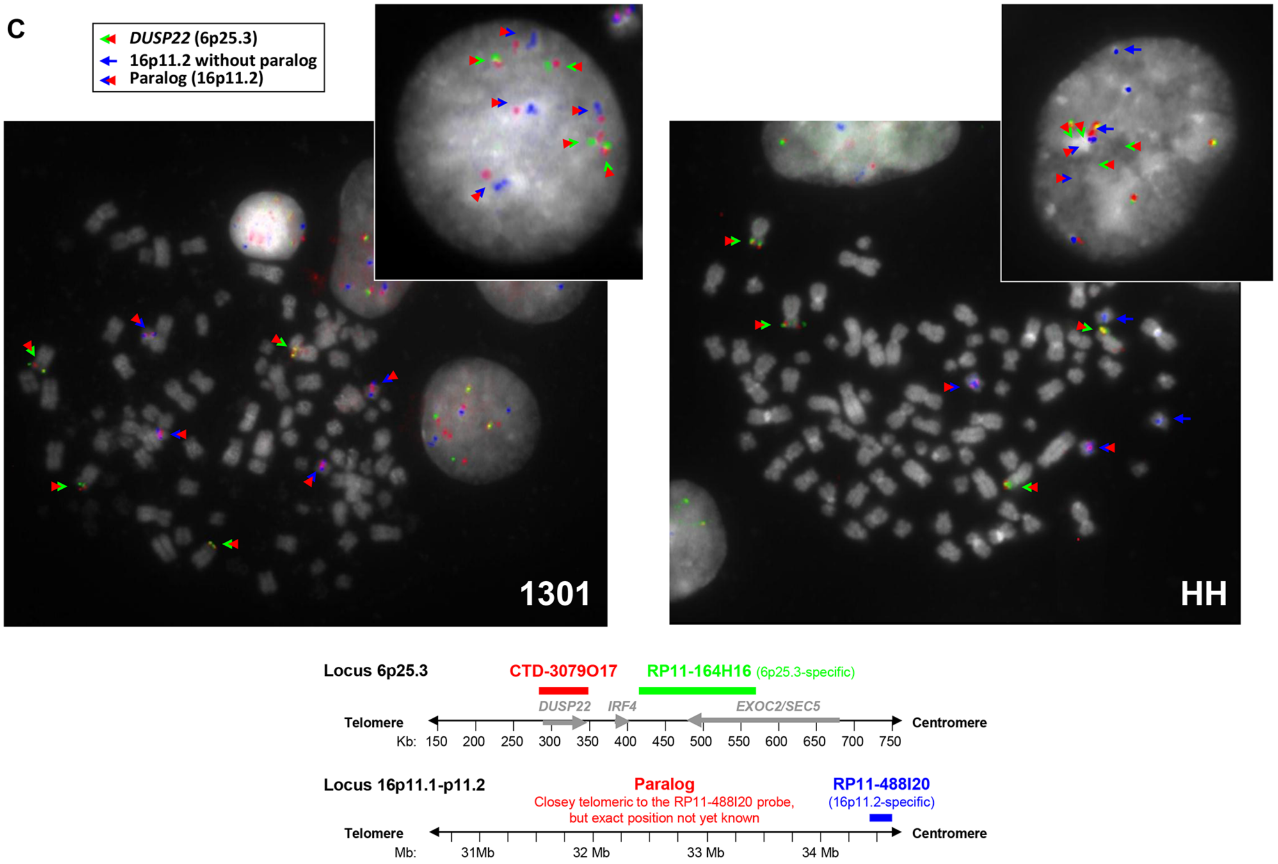

Supplementary Figure S7: Status of the 6p25.3 locus in lymphoid T-cell lines. C. Status of *DUSP22* and its paralog in 1301 and HH lymphoid T-cell lines, determined by FISH, as described in Figure 2. Results of metaphase and interphase (inserts) *FISH* analysis of 1301 and HH cells is shown, together with schematic representation of the 6p25.3 and 16p11.2 loci, including position of the genes and probes used for *FISH*. The probes used were RP11-164H16 (Spectrum Green labeled, specific of the 6p25.3 locus), CTD-3079O17 (Spectrum Red labeled, hybridizing on *DUSP22* and its paralog located on 16p11.2, when present; see Figure 2A, Supplementary Figures S5A, S5B, S6E and [31, 32]), and RP11-488I20 (Spectrum Gold labeled, here displayed in blue pseudocolor, as colocalization of signals from the former probes may appear in yellow, specific of the 16p11.2 locus). DAPI (4′,6-diamidino-2-phenylindole dihydrochloride), here visualized in grey (pseudo color), was used to stain nuclear DNA and chromosomes. Colocalization of green and red signals highlight the presence of *DUSP22* alleles, blue signals correspond to the 16p11.2 locus without paralog and colocalization of blue and red signals indicate the presence of 16p11.2 paralog copies, as indicated by uni- or bi-colored arrows. 1301 and HH cell lines both exhibit 4 copies of the *DUSP22* gene in most cells, without evidence of rearrangement or deletion, and respectively carry 4 and 2 copies of the 16p11.2 paralog (HH cells having also in general 2 copies of the 16p11.2 locus without paralog).

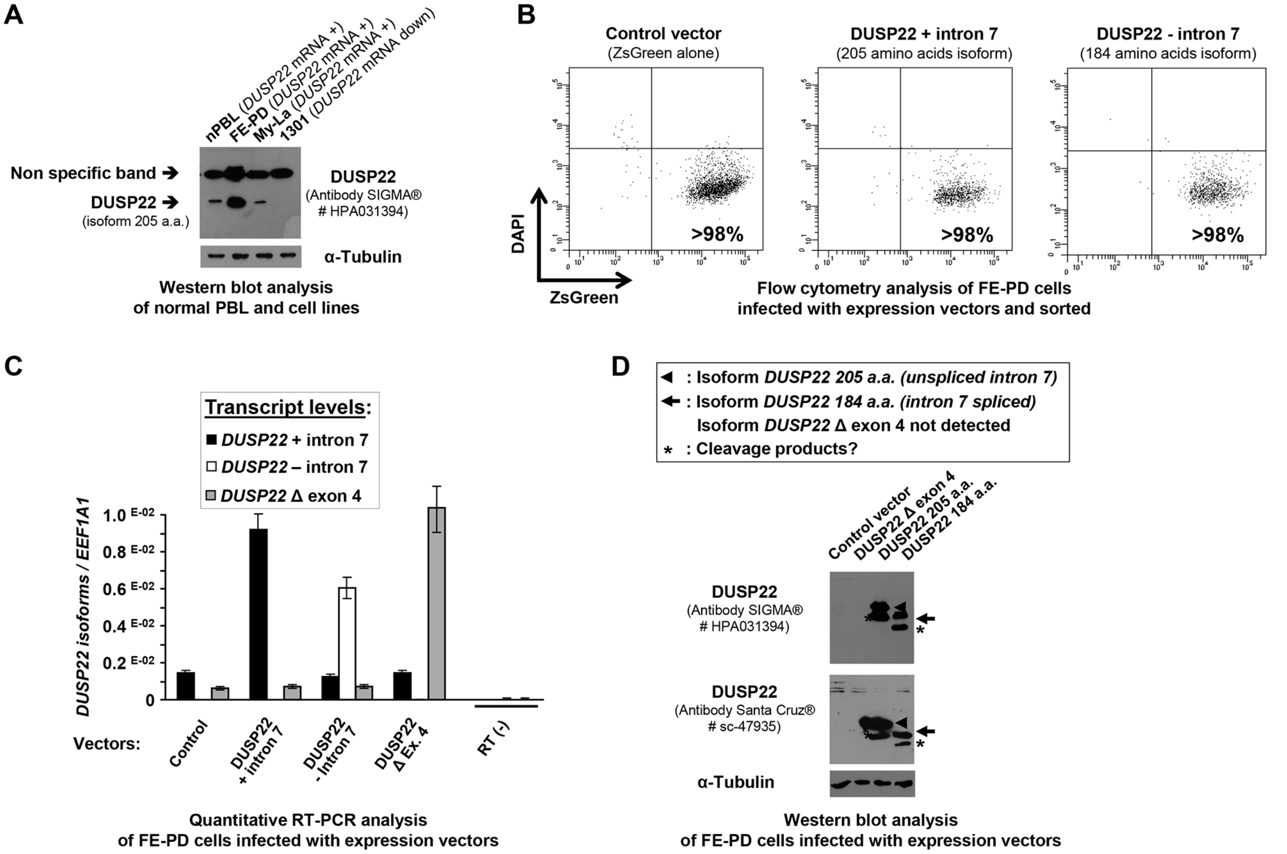

Supplementary Figure S8: Endogenous levels of DUSP22 protein in cell lines, cell sorting after lentiviral infection and analysis of ectopic expression of *DUSP22* isoforms in FE-PD ALCL cells. A. Western blot analysis of protein extracts from normal peripheral blood leukocytes (nPBL), T-cell lymphoma (My-La and FE-PD) and T-cell leukemia (1301) cell lines. A band was detected by the DUSP22 antibody used (HPA031394, from SIGMA®) in the molecular weight range compatible with DUSP22 isoforms. This band was undetectable in the 1301 cell line showing very low *DUSP22* transcript levels (Figures 1B, 1C). Nonetheless, the poor sensitivity and abundance of a higher molecular weight unspecific band hampers the use of this antibody for endogenous detection in most cell lines and also for *in situ* protein detection on cells slides and tissue sections. The other commercially available antibodies tested did not give better results (not shown). **B.** FE-PD cells were transduced with lentiviral vectors (Control -encoding the ZsGreen reporter alone- and bicistronic vectors encoding DUSP22 isoforms together with ZsGreen). Living (DAPI-) and transduced (ZsGreen+) cells were sorted by flow cytometry using ARIA II cell sorter. Dot plots show cell fraction purity after sorting. **C.** *DUSP22* isoforms transcript levels were analyzed by quantitative real-time reverse transcription-PCR (qRT-PCR) on RNA isolated from the same cells transduced with either control or DUSP22 isoforms expression vectors and sorted. *DUSP22* isoform-specific primers were used (Figure 1C and Supplementary Table S2) and transcript levels were normalized for *EEF1A1* gene expression and plotted at the y axis. Mean ± SEM from independent measurements are shown. **D.** Western blot analysis of DUSP22 expression in FE-PD cells infected with the control and DUSP22 isoforms vectors and sorted. The anti-DUSP22 antibodies used were from SIGMA® (# HPA031394, as in (**A**)) and Santa Cruz® (# sc-47935), the latter being directed against an N-terminal peptide common to the 3 isoforms. Alpha-tubulin (α-Tubulin) was used as a control for protein loading.

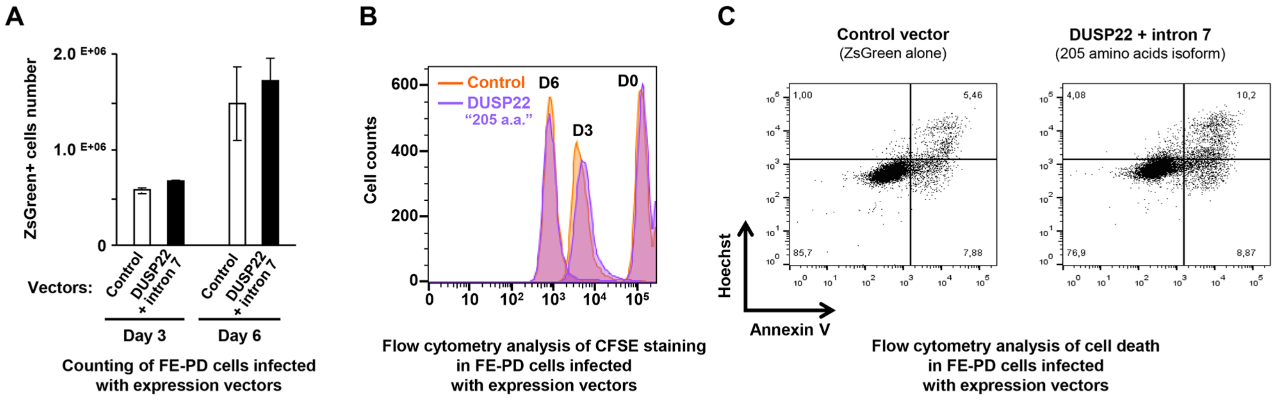

Supplementary Figure S9: Lack of impact of DUSP22 ectopic expression in DUSP22-expressing FE-PD cells. A. Effect of ectopic expression of the main DUSP22 isoform detected in lymphoid cells on proliferation and viability of FE-PD (cutaneous anaplastic large T cell lymphoma -ALCL-) cells. Cells infected with either the control (empty) or the DUSP22 205 amino acids (a.a.) isoform (Figure 1A and Supplementary Figure S1) lentiviral vectors and sorted were diluted in equal numbers and grown in the presence of 10% fetal calf serum and counted at different time points. Mean ± SEM from a representative experiment are shown. **B.** Proliferation assay of FE-PD cells transduced with either the control (empty) or the DUSP22 205 amino acids (a.a.) isoform lentiviral vectors and sorted. Cells were labeled with carboxyfluorescein succinimidyl ester (CFSE) at day-0 (D0) and its dilution was followed at day-3 (D3) and day-6 (D6) of culture. **C.** Apoptosis and necrosis analyses were performed on infected and sorted FE-PD cells by Annexin V/Hoescht 333542 staining 3 days after replating. Single Hoescht 333542-positivity indicates necrotic cells, while double Annexin V/Hoescht positivity hallmarks apoptotic cells.

Supplementary Table S1: Cutaneous T-cell lymphomas cases and T-lymphoid cell lines analyzed in our study, with histopathological and molecular features.

See Supplementary File 1

Supplementary Table S2: Primers (*F: forward; R: reverse*) used for quantitative PCR

Gene	Primers sequences (5'-3')	Location of primers and use
DUSP22-1F	AGCCACCATGGGGAATGGGAT	Exon 1 (cDNA, gDNA)
DUSP22-Int1R	CACCCCGGGCTCGAGTCGT	Intron 1 (gDNA, with 1F)
DUSP22-Ex3/4F	GGCCTATGTTGGAGGGAGTTA	Junction exon3-exon 4, transcripts with exon 4
DUSP22-Ex3/5F	GCCTATGTTGGAGGACAAGAC	Junction exon3-exon 5, Δ exon 4 transcripts
DUSP22-6F	GTGACACTGGTGATCGCATAC	Exon 6 (cDNA -all isoforms-)
DUSP22-6/7R	CTGCCGATACTGATGGACCT	Junction exon 7-exon 6 (cDNA -all isoforms-, gDNA)
DUSP22-6/Int6R2	AACTGCTTACCTGATGGACCT	Junction intron 6-exon 6 (gDNA, with 6F)
DUSP22-Ex7R	CGCCCTTGCTCCTTATATTTAC	Intron 7 (specific of transcripts with intron 7) isoform)
DUSP22-Ex8R	CATTACAGTCTTCTGAGAAAGG	Exon 8 (amplifies transcripts without intron 7)
IRF4-F	TGGCCAGAGGAAAAACATTGAG	Exon 9 (cDNA)
IRF4-R	TGTTGGGCCGGTGGTTGCACA	Exon 9 (cDNA)
EXOC2-F	GATCCTTCAGCTCATGCACA	Exon 13 (cDNA)
EXOC2-R	GACTGAGATGGCCCAACACT	Exon 14 (cDNA)
SFRP1-F	ATGCTTAAGTGTGACAAGTTCC	Exon 1 (cDNA)
SFRP1-R	TGGCCTCAGATTTCAACTCGT	Exon 2 (cDNA)
SFRP2-F	CATCCCCCTCGCTAGCAGC	Exon 1 (cDNA)
SFRP2-R	AAGCGTTTCCATTATGTCGTTGT	Exon 2 (cDNA)
EEF1A1-F	CTGGAGCCAAGTGCTAACATG	Exon 3 (cDNA)
EEF1A1-R	CCGGGTTTGAGAACACCAGT	Exon 4 (cDNA)
GAPDH-F	CTGCACCACCAACTGCTTAG	Exon 5 (cDNA)
GAPDH-R	AGGTCCACCACTGACACGTT	Exon 5 (cDNA)
TBP-F	AGTGAAGAACAGTCCAGACTG	Junction exons 6-7 (cDNA)
TBP-R	CCAGGAAATAACTCTGGCTCAT	Exon 8 (cDNA)
18S-F2	CCTGGATACCGCAGCTAGGA	*18S rRNA* (cDNA)
18S-R2	GCGGCGCAATACGAATGCCCC	*18S rRNA* (cDNA)
KLK3-F	AGGCTGGGGCAGCATTGAAC	Exon 3 (gDNA)
KLK3-R	CACCTTCTGAGGGTGAACTTG	Exon 4 (gDNA)

EEF1A1, *GAPDH*, *TBP* and *18S* rRNA were used as controls for normalization in quantitative reverse transcription (cDNA)-PCR analyses. *KLK3* was used as a control for normalization in quantitative genomic DNA (gDNA)-PCR analyses.

Supplementary Table S3: PCR Primers (*F: forward; R: reverse*) and methods used for SNP analyses

SNP analysis by sequencing

PCRs were performed from genomic DNA (gDNA) and cDNA, with primers listed bellow. PCR products were purified and sequenced by Sanger cycle sequencing using the forward primers for the sequencing reactions.

Gene	Primers sequences (5'-3')	Location of primers and usage
DUSP22-7F (rs11242812)	CAGGATGCAGAAGAAGCCAAAAAC	Exon 7; gDNA/cDNA PCR; sequencing
DUSP22-7Rbis (rs11242812)	TTAGGTCTCCGTCGTATAATTATCG	Intron 7; gDNA/cDNA PCR
DUSP22-8Fbis (rs1046656)	AGAGTTTAAGTATCCAGTAGTGAT	Exon 8 (3'UTR); gDNA/cDNA PCR; sequencing
DUSP22-SNP8R (rs1046656)	GCTGGGGAATTTGAGAACACAC	Exon 8 (3'UTR); gDNA/cDNA PCR

SNP analysis by digestion

PCRs were performed from genomic DNA (gDNA) and cDNA, with primers listed bellow. Digested PCR products were separated by 4% low melting agarose gel electrophoresis and visualized by SyBrGreen staining and UV illumination.

Gene	Primers sequences (5'-3')	Location of primers and usage
DUSP22-SNP7exFbis (rs11242812)	AGCCCGGCGCCAGGCGGTG	Intron 7; gDNA/cDNA PCR
DUSP22-SNPex7Rbis (rs11242812)	TTAGGTCTCCGTCGTATAATTATCG	Intron 7; gDNA/cDNA PCR
DUSP22-SNPex8F (rs1129085)	ACAAACCTCTCAGTGTATTCTTGG	Intron 7; gDNA PCR
DUSP22-Ex6/7F (rs1129085)	AGGTCCATCAGTATCGGCAG	Junction exon 6-exon 7; cDNA PCR
DUSP22-8R (rs1129085)	CATTACAGTCTTCTGAGAAAGG	Exon 8; gDNA/cDNA PCR
DUSP22-SNPex8Fbis (rs1046656)	AGAGTTTAAGTATCCAGTAGTGAT	Exon 8 (3'UTR); gDNA/cDNA PCR
DUSP22-SNPex8R (rs1046656)	GCTGGGGAATTTGAGAACACAC	Exon 8 (3'UTR); gDNA/cDNA PCR
SNP-IRF4-F (rs2316515)	AAACTTCCGTTCATTGCTCTC	Exon 11; gDNA/cDNA PCR
SNP-IRF4-R (rs2316515)	CGGGGCATGTAAAACATAACT	Exon 11; gDNA/cDNA PCR
SNP-EXOC2-F (rs11242914)	CACTTGCCCCACATTCACTG	Exon 28; gDNA/cDNA PCR
SNP-EXOC2-R (rs11242914)	CTGACAAGCCGACTACAAAGA	Exon 28; gDNA/cDNA PCR

(*Continued*)

Quantitative SNP alleles analysis

Real-time PCR was performed with a common forward primer and SNP allele-specific reverse primers (the 3' end nucleotide in bold is allele-specific, the underlined nucleotide being a deliberate mismatch introduced to enhance specificity). In parallel, PCRs were performed from the same DNA samples with *KLK3* primers (located on chromosome band 19q13.41 and used as a control gene).

To validate this real time PCR assay, PCR were performed with primers encompassing the SNP of interest, one of them being labeled at the 5' position by the 6-FAM fluorescent dye. PCR products were then digested with the *BssS*I restriction enzyme. All PCRs were performed with a number of cycles falling within the linear range of the reactions, as ascertained by real-time qPCR assays. Digested PCR products and *KLK3* control gene products were then pooled, denatured and analyzed by capillary electrophoresis on a Applied automated sequencer. Quantification was performed by measuring the area under curve and calculating ratios between peaks corresponding to SNP alleles and the control gene.

**Gene**	**Primers sequences (5'-3')**	**Location of primers and usage**
DUSP22-rs1046656(universal)-F	ACTGCTGTGGAGGTTTCTGTA	Exon 8 (3'UTR), real time, common to both alleles
DUSP22-rs1046656(C)-R3	CGTTCACAGGAAGCAAT<u>A</u>AC**G**	Exon 8 (3'UTR), real time, specific of allele "C"
DUSP22-rs1046656(T)-R3	CGTTCACAGGAAGCAAT<u>A</u>AC**A**	Exon 8 (3'UTR) real time, specific of allele "T"
DUSP22-8FbisN2 (rs1046656)	AGCCTTGCCGCACTGCCTTG	Exon 8 (3'UTR), electrophoresis
DUSP22-SNP8R (rs1046656)	GCTGGGGAATTTGAGAACACAC	Exon 8 (3'UTR) (6-FAM-labeled), electrophoresis
KLK3-F (control gene)	AGGCTGGGGCAGCATTGAAC	Exon 3 (6-FAM-labeled, except for real-time PCR)
KLK3-R (control gene)	CACCTTCTGAGGGTGAACTTG	Exon 4

Supplementary Table S4: PCR Primers (*F: forward; R: reverse*) used for methylation analyses

Gene	Primers sequences (5'-3')	Location of primers
**Methylation specific qPCR**		
DUSP22-1F-U (Unmethylated)	TGGGTTTTGAGATGAGTGTAGT	Proximal promoter
DUSP22-1R-U (Unmethylated)	CTAAACTCAAACTCCCAAACAA	Exon 1
DUSP22-1F-M (Methylated)	GTTTCGAGACGAGCGTAGTC	Proximal promoter
DUSP22-1R-M (Methylated)	ACTAAACTCGAACTCCCAAACG	Exon 1
IRF4-1F-U (Unmethylated)	AGTTTTTTTTGTATTAGATTTTTGT	Promoter
IRF4-1R-U (Unmethylated)	AACTATCACTAAAACCATTTCCCAC	Promoter
IRF4-1F-M (Methylated)	AGTTTTTTTCGTATTAGATTTTCGT	Promoter
IRF4-1R-M (Methylated)	CTATCACTAAAACCGTTTCCCG	Promoter
EXOC2-1F-U (Unmethylated)	TTGATTTTTAGTATTTGTGTTGG	Proximal promoter
EXOC2-1R-U (Unmethylated)	AAACAAAACCCAATAAATCTCACC	Exon 1
EXOC2-1F-M (Methylated)	TTGTTGATTTTTAGTATTCGCGTC	Proximal promoter
EXOC2-1R-M (Methylated)	AAACAAAACCCGATAAATCTCG	Exon 1
**Bisulfite sequencing**		
DUSP22-Bisulfite PCR-F	GTAGGGGAGTTTTTAGAGATTAGG	Proximal promoter (PCR)
DUSP22-Bisulfite PCR-R	AACAAACCCAACRTCCCTCCTC	Exon 1 (PCR)
DUSP22-Bisulfite PCR-Fbis	TTTTAGGGGAAAAGGTTAAAGGGG	Proximal promoter (Sequencing)

Supplementary Table S5: PCR Primers (*F: forward; R: reverse*) used for mutation analyses

High resolution melting and sequencing		
**Gene**	**Primers sequences (5'-3')**	**Location of primers**
DUSP22Ex1Fbis	CCATAGTGCGCCTGCGACC	Exon 1 (proximal 5'UTR, coding region, splice donor)
DUSP22-Int1R	CACCCCGGGCTCGAGTCGT	
DUSP22-Ex2Fbis	AGTCTGCCATGCTCATGTCTGT	Exon 2 (coding region and splice sites)
DUSP22-Ex2Rbis	CAACAATAAAAAAGCAAGAACTCAC	
DUSP22-Ex3F	CTGGCTAGACTTTAGGAGTAAT	Exon 3 (coding region and splice sites)
DUSP22-Ex3R	TACAAATGAGGACACCCACACA	
DUSP22-Ex4Fbis	CTTTTCTCCACTGAGTTTCATGTT	Exon 4 (coding region and splice sites)
DUSP22-Ex4Rbis	ATGTCTCCAAATAATACAGAAATAAG	
DUSP22-Ex5Fbis	GAGTAAAGTAGAGAGATGTCATTTC	Exon 5 (coding region and splice sites)
DUSP22-Ex5Rbis	AGGCGCTATTAAGCAAGAGAC	
DUSP22-Ex6F(int5)	ACCCGGAGATCTGAAACTGC	Exon 6 (coding region and splice sites)
DUSP22-Ex6R(int6)	GCACCTGCCTGCATCTCTG	
DUSP22-Ex7F(int6)	AAGCCCACGTGGATGCAGA	Exon 7 (coding region -including intron 7 splice variant- and splice sites)
DUSP22-Ex7R(int7)	AAGGCAGCAGGTCGCTTGC	
DUSP22-SNPex8F	CAAACCTCTCAGTGTATTCTTGG	Exon 8 (coding region and splice sites)
DUSP22-Ex8Rter	GTTGCTTTTCTTTTTGGCAGCA	

Supplementary Table S6: Summary of *DUSP22* SNPs genotyping and allelic expression, as well as methylation status and paralog copy number in normal lymphocytes and normal tissues.

See Supplementary File 2
